# Digital Emotion Regulation Interventions for Patients With Congenital Heart Disease: A Randomized Clinical Trial

**DOI:** 10.1001/jamanetworkopen.2025.38813

**Published:** 2025-10-24

**Authors:** Luise Pruessner, Steffen Hartmann, Anna-Lena Ehmann, Sven Barnow, Ulrike M. M. Bauer, Paul C. Helm

**Affiliations:** 1 Department of Psychology, Heidelberg University, Heidelberg, Germany; 2 Department of Congenital Heart Disease, Pediatric Cardiology, Deutsches Herzzentrum der Charité–Universitätsmedizin Berlin, Berlin, Germany; 3 National Register for Congenital Heart Defects, Berlin, Germany; 4 Competence Network for Congenital Heart Defects, Berlin, Germany

## Abstract

**Importance:**

Congenital heart disease (CHD) is associated with emotional distress and reduced quality of life, yet evidence-based psychosocial treatments are limited. Digital emotion regulation interventions may offer a scalable solution.

**Objective:**

To evaluate the effectiveness of tailored and general digital emotion regulation interventions compared with usual care for patients with CHD.

**Design, Setting, and Participants:**

This 3-arm, parallel-group randomized clinical trial was conducted from August 5, 2022, to October 21, 2024. Adults (≥18 years) with CHD were recruited nationwide in Germany.

**Interventions:**

Participants were randomized to a 4-week digital program with daily exercises to improve emotion regulation flexibility, delivered in a tailored or general format, or to usual CHD care.

**Main Outcomes and Measures:**

The primary outcome was change in emotion regulation difficulties. Secondary outcomes were the changes in emotion regulation repertoire, well-being, life satisfaction, anxiety, depression, stress, and illness identity. Analyses followed an intention-to-treat approach.

**Results:**

Of 1043 individuals screened, 524 were randomized (mean [SD] age, 35.2 [12.7] years; 364 women [69.5%]). At postintervention, the tailored program reduced emotion regulation difficulties compared with usual care (Cohen *d* = −0.45; 95% CI, −0.72 to −0.18; *P* = .001), whereas the general program did not. For secondary outcomes, the tailored program improved emotion regulation repertoire (Cohen *d* = 0.49; 95% CI, 0.18-0.78; *P* < .001), well-being (Cohen *d* = 0.39; 95% CI, 0.11-0.67; *P* = .006), life satisfaction (Cohen *d* = 0.37; 95% CI, 0.08-0.66; *P* = .01), anxiety (Cohen *d* = −0.38; 95% CI, −0.63 to −0.13; *P* = .003), depression (Cohen *d* = −0.40; 95% CI, −0.68 to −0.12; *P* = .005), and stress (Cohen *d* = −0.54; 95% CI, −0.86 to −0.22; *P* < .001), whereas the general program reduced stress only (Cohen *d* = −0.32; 95% CI, −0.58 to −0.05; *P* = .02). At follow-up, all primary and secondary gains for the tailored program were maintained, whereas the general program improved emotion regulation difficulties, anxiety, depression, and stress. Direct comparisons of the active interventions showed no difference in the primary outcome but greater gains in emotion regulation repertoire favoring the tailored program (postintervention: Cohen *d* = 0.26; 95% CI, 0.07-0.45; *P* = .007; follow-up: Cohen *d* = 0.33; 95% CI, 0.11-0.55; *P* = .004).

**Conclusions and Relevance:**

In this randomized clinical trial of adults with CHD, a tailored digital intervention improved emotion regulation and psychosocial outcomes at postintervention and at follow-up, whereas a general program showed more delayed benefits. These findings highlight the potential of tailored digital emotion regulation tools to meet the specific mental health needs of people living with chronic medical conditions.

**Trial Registration:**

ClinicalTrials.gov Identifier: NCT05862909

## Key Points

**Question:**

Can tailored or general digital emotion regulation interventions improve psychosocial outcomes among adults with congenital heart disease (CHD)?

**Findings:**

In this randomized clinical trial of 524 adults with CHD, a tailored digital intervention reduced emotion regulation difficulties vs usual care at postintervention and at follow-up and produced broader psychosocial gains at both assessments. A general digital intervention showed narrower benefits, differing from usual care primarily at follow-up.

**Meaning:**

These findings suggest that digital interventions have the potential to meet the mental health needs of individuals with CHD.

## Teaser

This randomized clinical trial evaluates tailored and general digital emotion regulation interventions compared with usual care for psychosocial outcomes among patients with congenital heart disease.

## Introduction

Congenital heart disease (CHD) affects an estimated 12 million people worldwide and 1.4 million adults in the US.^1,2,3^ Advances in cardiac surgery now allow most children with CHD to reach adulthood.^4,5^ Despite this progress, the psychosocial needs of this growing population remain largely unmet.^6,7,8^ Although half of adults with CHD meet the criteria for psychiatric disorders, fewer than 1 in 10 receive appropriate treatment.^8,9^ This gap is associated with poorer quality of life and increased clinical risk, highlighting the need for interventions that address the emotional health of this population.^10,11^

Patients with CHD face varying emotional challenges beginning in early life.^12,13,14,15,16^ These may include anxiety about medical procedures, social isolation from hospital stays, and frustration over activity restrictions in school, work, or social life.^11,17,18^ Collectively, these stressors contribute to rates of anxiety and depression that are 2-fold to 3-fold higher than in the general population.^9,10^ Without treatment, these difficulties can compound the disease burden for individuals and families and may impair adherence to medical care.^1,19,20,21,22^

Emotion regulation, the processes by which individuals modulate the onset, intensity, and expression of emotions, plays a central role in adapting to chronic illness.^23,24^ Recent models emphasize regulatory flexibility, defined as drawing on a repertoire of strategies and adjusting them according to contextual demands.^25,26,27^ For patients with CHD, strengthening both regulation abilities and the capacity for flexible adjustment may promote resilience, improve coping with uncertainty and recurrent medical stressors, and support long-term psychosocial well-being.^28,29,30^ Despite the importance of emotion regulation for mental health and quality of life, few interventions have directly targeted the emotional challenges of living with a complex congenital condition.^6,7,8,9^

Psychosocial interventions (eg, cognitive behavioral therapy and psychoeducation) have shown preliminary efficacy among individuals with CHD, particularly for reducing depressive symptoms.^31,32,33^ However, access to in-person care is often constrained by barriers such as limited mobility, geographic distance from specialized centers, and the cumulative burden of ongoing medical appointments.^34,35^ Digital interventions offer a promising alternative by providing scalable, personalized, and flexible support without additional clinic visits or stigma.^36,37,38,39^

To address this unmet need, this 3-arm randomized clinical trial tested a novel, tailored emotion regulation program and a general emotion regulation program against usual care and evaluated whether tailoring to the disease context conferred additional benefits. Both interventions were delivered digitally and designed to promote flexible emotion regulation by strengthening the sequence of identifying, selecting, implementing, and monitoring emotion regulation strategies.^40^ To capture broader psychosocial functioning, the trial examined effects on emotion regulation and various psychosocial outcomes, including well-being, life satisfaction, psychological distress, and illness identity.

## Methods

### Trial Design

This parallel-group, 3-arm, randomized clinical trial was conducted from August 5, 2022, to October 21, 2024, to evaluate the effectiveness of digital emotion regulation interventions for adults with CHD. The protocol was registered at ClinicalTrials.gov (Identifier: NCT05862909), approved by the institutional review boards of Heidelberg University and Charité–Universitätsmedizin Berlin, and conducted following the Declaration of Helsinki^41^ and the Consolidated Standards of Reporting Trials (CONSORT) reporting guideline. All participants provided written informed consent. The full trial protocol is available in Supplement 1.

### Participants

Participants were recruited from the National Register for Congenital Heart Defects, a nationwide German CHD registry.^42^ Adults aged 18 years or older with a clinically confirmed diagnosis of CHD were eligible for inclusion. Additional inclusion criteria were ownership of a smartphone with reliable internet access and advanced proficiency in German (Common European Framework of Reference for Languages level C1).

Exclusion criteria were incapacity to give informed consent, defined as the inability to understand the study information and risks or to make and communicate a voluntary decision, and acute suicidality, because digital interventions lack immediate clinical oversight for managing psychological crises.^43^ No further exclusion criteria were applied to ensure the study sample remained representative of routine clinical CHD populations.

### Randomization and Masking

After baseline, participants were randomized 1:1:1 to the tailored intervention, general intervention, or usual care using a computerized permuted-block algorithm.^44^ Allocation concealment was maintained through automated assignment via a secure online platform. All outcome questionnaires were pushed and collected through an automated platform to ensure unbiased data collection. Participants in the tailored and general intervention arms were blinded to the alternative intervention, and investigators analyzing outcome data were blinded to group allocations. All data were stored on secure servers in compliance with the EU General Data Protection Regulation.

### Interventions

Both intervention groups received a 4-week, self-directed digital program accessible via web browser and mobile app. Grounded in the extended process model of emotion regulation^40^ and contemporary models of emotion regulation flexibility,^25,26,27^ the interventions targeted the full regulatory sequence (identification, selection, implementation, and monitoring), emphasizing the ability to evaluate contextual demands, draw on a broad repertoire of strategies, and flexibly maintain, switch, or stop strategies as needed.^45,46,47^

The tailored program applied this framework to stressors unique to CHD, including anxiety related to medical procedures, frustration due to activity limitations, and health-related uncertainty.^48,49^ The general program delivered the same emotion regulation flexibility framework without CHD-specific contextualization.

The contents were adapted from validated cognitive behavioral protocols^50^ and structured as 10 sequenced video lessons with daily exercises over 4 weeks. Lessons 1 and 2 focused on identifying current emotions and regulatory goals,^51^ lessons 3 to 9 addressed the context-sensitive selection and implementation of 7 strategies (positive behavioral engagement, cognitive reappraisal, rumination reduction, acceptance, problem solving, avoidance reduction, and emotional expression), and lesson 10 emphasized monitoring and flexibly adjusting strategies to changing demands.^45,46,47^

To support transfer into daily life, the programs incorporated an app-based, just-in-time ecological momentary intervention,^52^ prompting in-the-moment emotion identification, contextual appraisal, goal setting, and strategy use. Adherence was monitored via automated logs of lesson and exercise completion. Additional details are provided in eTable 1 in Supplement 2.

### Usual Care Group

The usual care group did not receive access to the digital interventions during the trial but was granted full access to the tailored and general program after the final assessment. All participants continued their usual medical care for CHD throughout the study, including cardiology follow-up, diagnostic monitoring, and any indicated pharmacologic or psychological treatments.

### Outcome Measures

Data were collected at baseline; 4 weeks after randomization, corresponding to the end of the intervention (at postintervention); and 8 weeks after randomization (follow-up), to assess maintenance of effects. The primary outcome was change in emotion regulation difficulties (Difficulties in Emotion Regulation Scale)^53^ at the postintervention assessment. The primary comparison was the tailored intervention vs usual care. Prespecified secondary comparisons were general intervention vs usual care and tailored vs general intervention at postintervention, and all group contrasts at follow-up. Secondary outcome measures were emotion regulation repertoire (Heidelberg Form for Emotion Regulation Strategies),^54^ well-being (World Health Organization-Five Well-Being Index),^55^ life satisfaction (Satisfaction With Life Scale),^56^ depressive symptoms (Patient Health Questionnaire-9),^57^ anxiety symptoms (Generalized Anxiety Disorder Scale-7),^58^ perceived stress (Perceived Stress Scale),^59^ and illness identity (Illness Identity Questionnaire).^60^

### Statistical Analysis

Analyses followed an intention-to-treat approach and were performed in R, version 4.5.1 (R Project for Statistical Computing).^61^ We fitted linear mixed-effects models with random intercepts and fixed effects for group, time, and their interaction. *P* values for fixed-effect coefficients were obtained from Wald tests using the Satterthwaite degrees-of-freedom approximation; tests were 2-sided with α = .05. Cohen *d* effect sizes were calculated as the mean change divided by the pooled SD × √(2[1 − ρ]) for within-group changes^62^ and as the between-group difference in change scores divided by the pooled baseline SD.^63^ A priori power calculations in R (powerlmm; intraindividual correlation ρ = 0.40; α = .05; 80% power) yielded a target sample of at least 420 participants. Sensitivity analyses addressing missing data were performed using multiple imputation by chained equations (MICE). In addition, *P* values for secondary outcomes were adjusted for multiple comparisons using the Benjamini-Hochberg correction.^64^ Moderator analyses explored demographic and clinical characteristics. All procedures followed a prespecified analysis plan, with researchers blinded to group labels until after fitting the primary models.

## Results

### Participants

**Figure 1.**
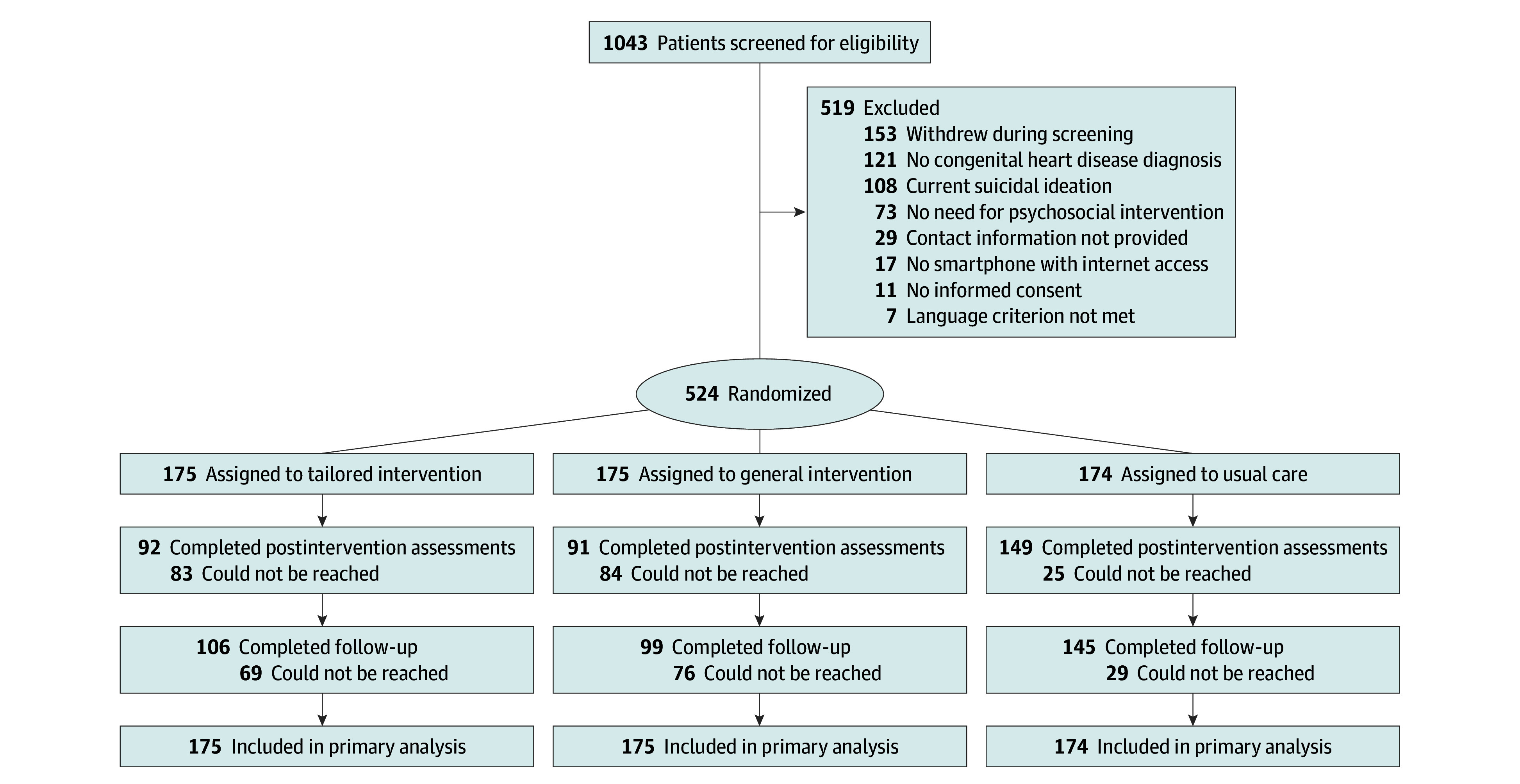
Participant Flow Diagram

Of 1043 patients initially screened, 524 were ultimately randomized (mean [SD] age, 35.2 [12.7] years; 364 women [69.5%] and 160 men [30.5%]) (Table 1). Participant enrollment and study flow are depicted in the CONSORT flow diagram (Figure 1). Table 1 describes baseline participant characteristics, illness severity, and descriptive statistics.

**Table 1.** Participants’ Demographic and Clinical Characteristics at Baseline

Characteristic	Participants, No. (%)			
	Total (N = 524)	Intervention groups		Usual care (n = 174)
		Tailored (n = 175)	General (n = 175)	
Age, mean (SD), y	35.2 (12.7)	35.2 (12.3)	35.2 (12.9)	35.2 (13.1)
Gender identity				
Male	160 (30.5)	53 (30.3)	50 (28.6)	57 (32.8)
Female	364 (69.5)	122 (69.7)	125 (71.4)	117 (67.2)
Nonbinary	0	0	0	0
Education				
Still in school	5 (1.0)	2 (1.1)	0	3 (1.7)
Primary school	29 (5.5)	11 (6.3)	10 (5.7)	8 (4.6)
Secondary school certificate	140 (26.7)	44 (25.1)	53 (30.3)	43 (24.7)
High school diploma	267 (51.0)	87 (49.7)	84 (48.0)	96 (55.2)
Vocational school certificate	75 (14.3)	28 (16.0)	27 (15.4)	20 (11.5)
Other	8 (1.5)	3 (1.7)	1 (0.6)	4 (2.3)
Nationality				
German	517 (98.7)	174 (99.4)	174 (99.4)	169 (97.1)
Other	7 (1.3)	1 (0.6)	1 (0.6)	5 (2.9)
CHD severity^a^				
Simple	64 (12.2)	23 (13.1)	19 (10.9)	22 (12.6)
Moderate	263 (50.2)	88 (50.3)	88 (50.3)	87 (50.0)
Complex	154 (29.4)	54 (30.9)	53 (30.3)	47 (27.0)
Unclassifiable	43 (8.2)	10 (5.7)	15 (8.6)	18 (10.3)
Type of CHD diagnosis				
Transposition with intact septum	41 (7.8)	17 (9.7)	11 (6.3)	13 (7.5)
Complex transposition	9 (1.7)	3 (1.7)	2 (1.1)	4 (2.3)
Corrected transposition	17 (3.2)	7 (4.0)	6 (3.4)	4 (2.3)
Aortic coarctation	43 (8.2)	11 (6.3)	13 (7.4)	19 (10.9)
Pulmonary atresia	10 (1.9)	3 (1.7)	3 (1.7)	4 (2.3)
Patent ductus arteriosus	13 (2.5)	6 (3.4)	6 (3.4)	1 (0.6)
Ventricular septal defect	76 (14.5)	29 (16.6)	31 (17.7)	16 (9.2)
Atrial septal defect	8 (1.5)	1 (0.6)	3 (1.7)	4 (2.3)
Tetralogy of Fallot	52 (9.9)	20 (11.4)	16 (9.1)	16 (9.2)
Ebstein anomaly	52 (9.9)	16 (9.1)	16 (9.1)	20 (11.5)
Cardiomyopathy	11 (2.1)	6 (3.4)	2 (1.1)	3 (1.7)
Univentricular heart	52 (9.9)	12 (6.9)	23 (13.1)	17 (9.8)
Coronary artery anomaly	5 (1.0)	1 (0.6)	2 (1.1)	2 (1.1)
Truncus arteriosus	4 (0.8)	2 (1.1)	0	2 (1.1)
Atrioventricular septal defect	18 (3.4)	10 (5.7)	5 (2.9)	3 (1.7)
Partial anomalous pulmonary venous connection	7 (1.3)	1 (0.6)	4 (2.3)	2 (1.1)
Total anomalous pulmonary venous connection	2 (0.4)	2 (1.1)	0	0
Pulmonary artery anomaly	42 (8.0)	11 (6.3)	12 (6.9)	19 (10.9)
Aortic valve anomaly	8 (1.5)	1 (0.6)	1 (0.6)	6 (3.4)
Patent foramen ovale	2 (0.4)	0	1 (0.6)	1 (0.6)
Shone complex	3 (0.6)	0	1 (0.6)	2 (1.1)
Interrupted aortic arch	1 (0.2)	0	0	1 (0.6)
Marfan syndrome	4 (0.8)	3 (1.7)	0	1 (0.6)
Other heart defects	44 (8.4)	13 (7.4)	17 (9.7)	14 (8.0)
No. of heart surgical procedures				
None	120 (22.9)	39 (22.3)	44 (25.1)	37 (21.3)
1	190 (36.3)	64 (36.6)	60 (34.3)	66 (37.9)
2	106 (20.2)	32 (18.3)	36 (20.6)	38 (21.8)
3	61 (11.6)	26 (14.9)	18 (10.3)	17 (9.8)
≥4	47 (9.0)	14 (8.0)	17 (9.7)	16 (9.2)
Medical comorbidities				
Pulmonary disease	37 (7.1)	14 (8.0)	9 (5.1)	14 (8.0)
Digestive disorder	17 (3.2)	6 (3.4)	5 (2.9)	6 (3.4)
Neurologic disorder	9 (1.7)	2 (1.1)	5 (2.9)	2 (1.1)
Immune system disorder	15 (2.9)	3 (1.7)	5 (2.9)	7 (4.0)
Kidney disease	17 (3.2)	2 (1.1)	8 (4.6)	7 (4.0)
Liver disease	17 (3.2)	4 (2.3)	9 (5.1)	4 (2.3)
Cancer	5 (1.0)	1 (0.6)	3 (1.7)	1 (0.6)
Other diseases	92 (17.6)	30 (17.1)	35 (20.0)	27 (15.5)
Current psychotherapy	73 (13.9)	30 (17.1)	24 (13.7)	19 (10.9)
Current psychopharmacotherapy	53 (10.1)	14 (8.0)	23 (13.1)	16 (9.2)
Psychiatric comorbidities				
Depressive disorder	114 (21.8)	40 (22.9)	36 (20.6)	38 (21.7)
Anxiety disorder	93 (17.7)	27 (15.4)	36 (20.6)	30 (17.2)
Eating disorder	13 (2.5)	3 (1.7)	3 (1.7)	7 (4.1)
Somatoform disorder	6 (1.1)	1 (0.6)	3 (1.7)	2 (1.1)
Posttraumatic stress disorder	38 (7.2)	19 (10.9)	12 (6.9)	7 (4.0)
Bipolar disorder	2 (0.4)	0	2 (1.1)	0
Borderline personality disorder	6 (1.1)	3 (1.7)	3 (1.7)	0
Other personality disorder	3 (0.6)	0	1 (0.6)	2 (1.1)
Attention-deficit/hyperactivity disorder	12 (2.3)	2 (1.1)	5 (2.9)	5 (2.9)
Psychosis	4 (0.8)	1 (0.6)	2 (1.1)	1 (0.6)
Substance use disorder	4 (0.8)	0	3 (1.7)	1 (0.6)

Abbreviation: CHD, congenital heart disease.

^a^ CHD severity is categorized into 3 levels (simple, moderate, and complex) based on anatomical complexity, clinical presentation, and the need for intervention.

### Changes in the Primary Outcome

**Figure 2.**
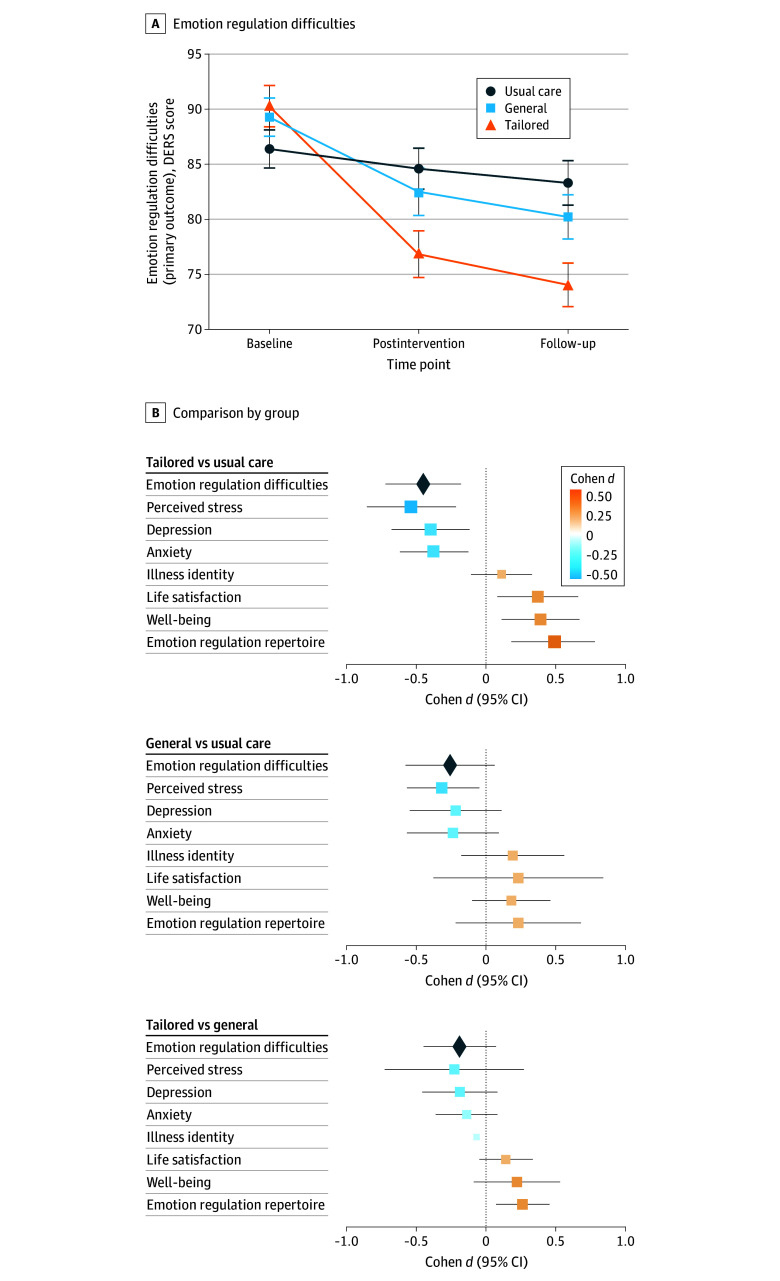
Observed Trajectories in Emotion Regulation Difficulties and Between-Group Differences Across Outcomes Forest plots show between-group differences at the postintervention assessment (Cohen *d* with 95% CIs). The primary outcome (emotion regulation difficulties) is indicated by a diamond. Squares indicate secondary outcomes. DERS indicates Difficulties in Emotion Regulation Scale (score range, 36-180; higher scores indicate greater difficulties in emotion regulation).

From baseline to postintervention, difficulties in emotion regulation decreased in the tailored program (mean change, −12.36; Cohen *d* = −0.67; 95% CI, −1.07 to −0.27; *P* < .001) and the general program (mean change, −7.41; Cohen *d* = −0.43; 95% CI, −0.73 to −0.13; *P* = .005) but not in the usual care group (mean change, −1.88; Cohen *d* = −0.10; 95% CI, −0.21 to 0.00; *P* = .06) (Table 2^53,54,55,56,57,58,59,60^; Figure 2). From baseline to follow-up, decreases in emotion regulation difficulties persisted.

**Table 2.** Changes in Primary and Secondary Outcomes From Baseline to Postintervention and Follow-Up^a^

Outcome and group	Mean (SD) score			Postintervention changes^b^			Follow-up changes^b^		
	Baseline	Postintervention	Follow-up	Cohen *d* (95% CI)	*P* value	Cohen *d* (95% CI)		*P* value
**Primary outcome**									
Difficulties in emotion regulation (DERS)^53^								
Tailored	90.33 (24.87)	77.97 (21.02)	75.71 (19.86)	−0.67 (−1.07 to −0.27)	<.001	−0.82 (−1.03 to −0.60)		<.001
General	89.25 (23.16)	81.84 (20.24)	79.63 (20.63)	−0.43 (−0.73 to −0.13)	.005	−0.55 (−0.74 to −0.36)		<.001
Usual care	86.33 (22.94)	84.45 (22.41)	83.12 (24.02)	−0.10 (−0.21 to 0.00)	.06	−0.17 (−0.31 to −0.03)		.008
**Secondary outcomes**									
Emotion regulation repertoire (HFERST)^54^								
Tailored	9.80 (2.13)	10.85 (1.89)	10.85 (1.73)	0.64 (0.26 to 1.02)	<.001	0.67 (0.44 to 0.89)		<.001
General	10.29 (1.95)	10.79 (1.86)	10.69 (1.83)	0.32 (−0.01 to 0.66)	.06	0.26 (0.01 to 0.51)		.05
Usual care	10.02 (1.77)	10.08 (1.79)	10.08 (1.89)	0.04 (−0.02 to 0.11)	.21	0.04 (−0.09 to 0.17)		.31
Well-being (WHO-5)^55^								
Tailored	50.61 (19.56)	55.09 (17.75)	55.55 (17.87)	0.30 (−0.01 to 0.62)	.06	0.33 (0.11 to 0.56)		.03
General	51.54 (21.42)	52.00 (20.14)	51.52 (19.30)	0.03 (−0.21 to 0.27)	.82	−0.001 (−0.21 to 0.21)		.79
Usual care	52.21 (20.27)	49.07 (20.40)	50.95 (20.31)	−0.19 (−0.38 to −0.01)	.04	−0.08 (−0.29 to 0.14)		.31
Life satisfaction (SWLS)^56^								
Tailored	23.87 (6.38)	26.04 (5.40)	25.95 (5.52)	0.65 (0.26 to 1.03)	.001	0.61 (0.42 to 0.81)		.001
General	24.22 (6.61)	25.57 (5.29)	24.74 (6.40)	0.40 (−0.25 to 1.04)	.23	0.14 (−0.12 to 0.40)		.26
Usual care	24.13 (6.00)	24.12 (6.14)	24.51 (6.15)	−0.003 (−0.02 to 0.01)	.65	0.11 (−0.05 to 0.27)		.18
Anxiety (GAD-7)^58^								
Tailored	7.27 (4.63)	5.80 (3.86)	5.82 (4.22)	−0.46 (−0.73 to −0.18)	.001	−0.43 (−0.68 to −0.19)		.001
General	6.86 (4.68)	5.98 (3.88)	5.83 (3.68)	−0.27 (−0.59 to 0.05)	.10	−0.32 (−0.55 to −0.10)		.002
Usual care	6.33 (3.97)	6.46 (4.18)	6.57 (4.45)	0.04 (−0.69 to 0.77)	.91	0.08 (−0.09 to 0.24)		.80
Depression (PHQ-9)^57^								
Tailored	8.14 (4.73)	6.68 (4.16)	6.85 (4.14)	−0.47 (−0.78 to −0.16)	.003	−0.42 (−0.63 to −0.21)		.005
General	7.79 (4.76)	7.16 (4.10)	7.13 (4.01)	−0.20 (−0.59 to 0.18)	.30	−0.22 (−0.47 to 0.03)		.06
Usual care	7.31 (4.21)	7.64 (4.43)	7.61 (4.47)	0.11 (−0.16 to 0.38)	.42	0.10 (−0.09 to 0.29)		.41
Perceived stress (PSS)^59^								
Tailored	10.66 (3.27)	9.24 (2.49)	9.42 (2.76)	−0.70 (−1.11 to −0.28)	<.001	−0.59 (−0.86 to −0.31)		.001
General	10.68 (3.28)	9.90 (2.61)	10.07 (2.81)	−0.38 (−0.66 to −0.09)	.01	−0.29 (−0.55 to −0.02)		.01
Usual care	10.63 (2.95)	10.80 (3.14)	10.66 (3.14)	0.08 (−0.61 to 0.77)	.82	0.01 (−0.19 to 0.22)		.81
Illness identity (IIQ)^60^								
Tailored	3.01 (0.26)	3.05 (0.26)	3.07 (0.26)	0.18 (−0.04 to 0.40)	.11	0.27 (0.08 to 0.46)		.01
General	3.03 (0.26)	3.09 (0.27)	3.10 (0.24)	0.26 (−0.03 to 0.56)	.08	0.33 (0.11 to 0.54)		.007
Usual care	3.02 (0.26)	3.03 (0.25)	3.03 (0.27)	0.05 (−0.08 to 0.17)	.46	0.04 (−0.10 to 0.19)		.40

Abbreviations: DERS, Difficulties in Emotion Regulation Scale (score range, 36-180; higher scores indicate more emotion regulation difficulties); GAD-7, Generalized Anxiety Disorder Scale-7 (score range, 0-21; higher scores indicate more severe anxiety); HFERST, Heidelberg Form for Emotion Regulation Strategies (score range, 3-15; the emotion regulation repertoire was operationalized as the sum of cognitive reappraisal, acceptance, and problem solving reported by participants, with higher scores indicating a broader repertoire); IIQ, Illness Identity Questionnaire (score range, 1-5; composite coded so higher scores indicate more adaptive illness identity); PHQ-9, Patient Health Questionnaire-9 (score range, 0-27; higher scores indicate more severe depression); PSS, Perceived Stress Scale (score range, 0-16; higher scores indicate greater perceived stress); SWLS, Satisfaction With Life Scale (score range, 5-35; higher scores indicate greater life satisfaction); WHO-5, World Health Organization-Five Well-Being Index (score range, 0-100; higher scores indicate better well-being).

^a^ All analyses were conducted in the full intent-to-treat cohort (N = 524; tailored, n = 175; general, n = 175; usual care, n = 174).

^b^ Cohen *d* values reflect within-group standardized mean changes from baseline, calculated using pooled baseline SDs. *P* values are unadjusted (2-sided tests; α = .05).

In mixed-effects models testing group-by-time interaction effects, the tailored intervention showed greater reductions in emotion regulation difficulties than the usual care group at postintervention (Cohen *d* = −0.45; 95% CI, −0.72 to −0.18; *P* = .001) and at follow-up (Cohen *d* = −0.51; 95% CI, −0.81 to −0.20; *P* < .001) (Table 3).^53,54,55,56,57,58,59,60^ The general intervention differed from the usual care group at follow-up only (Cohen *d* = −0.30; 95% CI, −0.50 to −0.10; *P* = .003). No significant differences were observed between the tailored and general interventions at either time point.

**Table 3.** Mixed Models of Group-by-Time Interaction Effects in Primary and Secondary Outcomes at Postintervention and at Follow-Up^a^

Outcome and group comparison	Postintervention effects^b^			Follow-up effects^b^		
	*b* (SE)	Cohen *d* (95% CI)	*P* value	*b* (SE)	Cohen *d* (95% CI)	*P* value
**Primary outcome**						
Difficulties in emotion regulation (DERS)^53^						
Tailored vs usual care	−6.76 (2.09)	−0.45 (−0.72 to −0.18)	.001	−8.87 (2.28)	−0.51 (−0.81 to −0.20)	<.001
General vs usual care	−3.28 (2.06)	−0.26 (−0.58 to 0.06)	.11	−6.50 (2.19)	−0.30 (−0.50 to −0.10)	.003
Tailored vs general	−3.79 (2.61)	−0.19 (−0.45 to 0.07)	.15	−2.89 (2.70)	−0.21 (−0.60 to 0.18)	.29
**Secondary outcomes**						
Emotion regulation repertoire (HFERST)^54^						
Tailored vs usual care	0.87 (0.18)	0.49 (0.18 to 0.78)	<.001	1.02 (0.19)	0.51 (0.18 to 0.82)	<.001
General vs usual care	0.19 (0.19)	0.23 (−0.22 to 0.68)	.31	0.30 (0.20)	0.18 (−0.06 to 0.42)	.13
Tailored vs general	0.68 (0.25)	0.26 (0.07 to 0.45)	.007	0.76 (0.26)	0.33 (0.11 to 0.55)	.004
Well-being (WHO-5)^55^						
Tailored vs usual care	5.91 (2.13)	0.39 (0.11 to 0.67)	.006	5.51 (2.41)	0.33 (0.05 to 0.61)	.02
General vs usual care	2.68 (2.12)	0.18 (−0.10 to 0.46)	.21	1.20 (2.45)	0.06 (−0.18 to 0.30)	.63
Tailored vs general	3.04 (2.15)	0.22 (−0.09 to 0.53)	.16	4.06 (2.44)	0.26 (−0.05 to 0.57)	.10
Life satisfaction (SWLS)^56^						
Tailored vs usual care	1.09 (0.43)	0.37 (0.08 to 0.66)	.01	0.85 (0.43)	0.29 (0.01 to 0.58)	.049
General vs usual care	0.32 (0.43)	0.23 (−0.38 to 0.84)	.46	0.15 (0.49)	0.02 (−0.11 to 0.15)	.76
Tailored vs general	0.77 (0.52)	0.14 (−0.05 to 0.33)	.14	0.76 (0.57)	0.27 (−0.13 to 0.67)	.18
Anxiety (GAD-7)^58^						
Tailored vs usual care	−1.24 (0.42)	−0.38 (−0.63 to −0.13)	.003	−1.51 (0.48)	−0.38 (−0.62 to −0.14)	.002
General vs usual care	−0.60 (0.42)	−0.24 (−0.57 to 0.09)	.16	−1.23 (0.46)	−0.30 (−0.52 to −0.08)	.007
Tailored vs general	−0.65 (0.53)	−0.14 (−0.36 to 0.08)	.22	−0.28 (0.56)	−0.08 (−0.39 to 0.23)	.62
Depression (PHQ-9)^57^						
Tailored vs usual care	−1.29 (0.45)	−0.40 (−0.68 to −0.12)	.005	−1.25 (0.46)	−0.36 (−0.62 to −0.10)	.007
General vs usual care	−0.60 (0.46)	−0.22 (−0.55 to 0.11)	.19	−1.00 (0.49)	−0.22 (−0.43 to −0.01)	.04
Tailored vs general	−0.67 (0.48)	−0.19 (−0.46 to 0.08)	.17	−0.24 (0.51)	−0.14 (−0.72 to 0.44)	.64
Perceived stress (PSS)^59^						
Tailored vs usual care	−0.58 (0.17)	−0.54 (−0.86 to −0.22)	<.001	−0.45 (0.18)	−0.42 (−0.75 to −0.09)	.01
General vs usual care	−0.42 (0.17)	−0.32 (−0.58 to −0.05)	.02	−0.35 (0.18)	−0.21 (−0.42 to −0.001)	.049
Tailored vs general	−0.37 (0.41)	−0.23 (−0.73 to 0.27)	.36	−0.23 (0.41)	−0.21 (−0.94 to 0.52)	.57
Illness identity (IIQ)^60^						
Tailored vs usual care	0.03 (0.03)	0.11 (−0.11 to 0.33)	.28	0.04 (0.03)	0.19 (−0.09 to 0.47)	.10
General vs usual care	0.03 (0.03)	0.19 (−0.18 to 0.56)	.29	0.06 (0.03)	0.24 (−0.01 to 0.48)	.06
Tailored vs general	−0.01 (0.04)	−0.07 (−0.62 to 0.48)	.89	−0.01 (0.03)	−0.05 (−0.34 to 0.25)	.75

Abbreviations: DERS, Difficulties in Emotion Regulation Scale; GAD-7, Generalized Anxiety Disorder Scale-7; HFERST, Heidelberg Form for Emotion Regulation Strategies (the emotion regulation repertoire was operationalized as the sum of cognitive reappraisal, acceptance, and problem solving reported by participants, with higher scores indicating a broader repertoire); IIQ, Illness Identity Questionnaire; PHQ-9, Patient Health Questionnaire-9; PSS, Perceived Stress Scale; SWLS, Satisfaction With Life Scale; WHO-5, World Health Organization-Five Well-Being Index.

^a^ All analyses were conducted in the full intent-to-treat cohort (N = 524; tailored, n = 175; general, n = 175; usual care, n = 174); pairwise comparisons are based on the respective group sizes within the model.

^b^ Cohen *d* values reflect between-group differences in score changes, calculated using pooled baseline SDs. *P* values are unadjusted (2-sided tests; α = .05). False discovery rate–adjusted *P* values for secondary outcomes are provided in eTable 8 in Supplement 2. Interpretation remained consistent after correction, except for the analyses of the general intervention vs usual care for stress and depression.

### Secondary Outcomes

#### Emotion Regulation Repertoire

Participants in the tailored group showed significant improvements in their emotion regulation repertoire, reflecting the range of regulatory strategies a person can access and flexibly use across emotional contexts. Compared with usual care, gains were greater both at postintervention (Cohen *d* = 0.49; 95% CI, 0.18-0.78; *P* < .001) and at follow-up (Cohen *d* = 0.51; 95% CI, 0.18-0.82; *P* < .001) (Table 3). The tailored group also outperformed the general intervention group at postintervention (Cohen *d* = 0.26; 95% CI, 0.07-0.45; *P* = .007) and at follow-up (Cohen *d* = 0.33; 95% CI, 0.11-0.55; *P* = .004). No significant change was observed in the general intervention group relative to the usual care group. Effects for specific emotion regulation strategies are provided in eTable 2 in Supplement 2.

#### Well-Being and Life Satisfaction

Subjective well-being improved in the tailored group, with greater improvement than in the usual care group both at postintervention (Cohen *d* = 0.39; 95% CI, 0.11-0.67; *P* = .006) and at follow-up (Cohen *d* = 0.33; 95% CI, 0.05-0.61; *P* = .02) (Table 3). Life satisfaction also increased in the tailored program, with interaction effects favoring the tailored group over the usual care group at both time points (postintervention: Cohen *d* = 0.37; 95% CI, 0.08-0.66; *P* = .01; follow-up: Cohen *d* = 0.29; 95% CI, 0.01-0.58; *P* = .049). The general intervention group did not differ significantly from the usual care group regarding either outcome.

#### Anxiety, Depression, and Perceived Stress

Participants in the tailored intervention group reported significantly greater decreases in anxiety than the usual care group at postintervention (Cohen *d* = −0.38; 95% CI, −0.63 to −0.13; *P* = .003) and at follow-up (Cohen *d* = −0.38; 95% CI, −0.62 to −0.14; *P* = .002) (Table 3). The general intervention group showed anxiety reductions vs the usual care group only at follow-up (Cohen *d* = −0.30; 95% CI, −0.52 to −0.08; *P* = .007).

Depressive symptoms also decreased in the tailored intervention group, with larger changes compared with the usual care group at postintervention (Cohen *d* = −0.40; 95% CI, −0.68 to −0.12; *P* = .005) and at follow-up (Cohen *d* = −0.36; 95% CI, −0.62 to −0.10; *P* = .007) (Table 3). The general intervention group differed from the usual care group only at follow-up (Cohen *d* = −0.22; 95% CI, −0.43 to −0.01; *P* = .04).

Perceived stress decreased significantly in the tailored intervention group compared with the usual care group at postintervention (Cohen *d* = −0.54; 95% CI, −0.86 to −0.22; *P* < .001) and at follow-up (Cohen *d* = −0.42; 95% CI, −0.75 to −0.09; *P* = .01) (Table 3). The general intervention group also showed significant reductions in perceived stress vs the usual care group at postintervention (Cohen *d* = −0.32; 95% CI, −0.58 to −0.05; *P* = .02) and at follow-up (Cohen *d* = −0.21; 95% CI, −0.42 to −0.01; *P* = .049).

#### Illness Identity

In mixed-effects models, neither intervention differed significantly from usual care on illness identity at postintervention or at follow-up. Similar findings for individual illness identity components are provided in eTable 3 in Supplement 2.

### Attrition, Adherence, and Adverse Effects

Attrition was defined as missing either the postintervention or follow-up assessment; intervention adherence was defined as the number of completed video lessons and exercises, as recorded by automated platform logs. In multivariable logistic regression models, only female gender was associated with lower odds of attrition (odds ratio, 0.59; 95% CI, 0.40-0.88; *P* = .009) (eTable 4 in Supplement 2).

Baseline characteristics were not significantly associated with adherence. However, adherence differed by group; those in the tailored program completed more lessons and exercises than those in the general program (mean [SE] difference, 2.00 [0.75] exercises; 95% CI, 0.55-3.24; *P* = .007). Across both groups, greater adherence was associated with larger treatment effects (eTable 4 in Supplement 2).

No serious treatment-related adverse events occurred during the intervention or at follow-up. At postintervention, 183 participants across both intervention arms completed the Negative Effects Questionnaire^65^; 8 participants (4.4%) reported transient negative effects, most commonly stress, negative emotions, or resurfacing memories. No negative effects were reported at follow-up (eTable 5 in Supplement 2).

### Sensitivity and Moderator Analyses

Sensitivity analyses using MICE confirmed the primary findings (eTables 6 and 7 in Supplement 2), suggesting that the conclusions are not driven by selective attrition. For secondary outcomes, the effects of the general intervention vs usual care on depression and perceived stress did not remain statistically significant after correction for multiple testing, whereas all other findings were unchanged (eTable 8 in Supplement 2).

Moderator analyses indicated that participants with higher baseline stress or anxiety experienced significantly greater improvements in both the tailored vs usual care and general vs usual care comparisons. Conversely, higher baseline life satisfaction was associated with significantly smaller improvements in both the tailored vs usual care and general vs usual care analyses. No moderation was seen for age, gender, educational level, psychotherapy, CHD severity, surgery count, or the number of mental disorders (eTable 9 in Supplement 2).

## Discussion

Patients with CHD experience persistent emotional distress, including elevated anxiety and depression,^10,66^ yet scalable psychosocial interventions remain limited.^31^ To our knowledge, this randomized clinical trial is the first to test fully digital emotion regulation interventions in this population. The programs, grounded in models of emotion regulation flexibility,^25^ combined sequenced lessons with app-based practice to target key stages of emotion regulation, from identifying emotions to monitoring regulatory success.^40^ Compared with usual care, both interventions improved emotion regulation difficulties at follow-up, while the tailored program produced earlier and broader benefits across secondary outcomes, including emotion regulation repertoire, well-being, and life satisfaction.

In addition to demonstrating effectiveness relative to usual care, the trial also addressed whether tailoring the digital intervention to disease context confers advantages over a general program. Although between-group differences were modest on the primary outcome, the tailored program showed larger effect sizes, higher adherence, and broader secondary gains. Several factors may explain why contrasts between active interventions emerged mainly on secondary outcomes; global regulation difficulties may be less sensitive to CHD-specific mechanisms, and both programs incorporated overlapping evidence-based components (eg, emotional awareness and monitoring emotion regulation success), which may have driven similar improvements on global outcomes. These findings suggest tailoring may enhance benefits but also show that general programs provide meaningful support, an important consideration given gaps in psychosocial care for adults with CHD.^12,13,14,15^

Consistent with recent theoretical models, the results indicate that enhancing emotion regulation skills and their flexible, context-sensitive use is associated with benefits in multiple domains of functioning, including anxiety, depression, and stress.^26,45,46,47,51^ This observation is also in line with prior studies in healthy populations^67^ and among individuals with mental disorders,^68^ demonstrating that digital interventions can enhance emotion regulation abilities and, in turn, broader indicators of psychological well-being.

Moderator analyses identified subgroups most likely to benefit from the digital intervention programs. Participants with higher baseline emotional distress and lower life satisfaction experienced the most pronounced improvements. These findings highlight the potential of tailored digital interventions to reach individuals with unmet mental health needs, especially in underserved populations. In this context, digital tools may complement traditional psychotherapy by offering timely, scalable support.^69,70^

Despite improvements across various psychological outcomes, the interventions did not significantly alter illness identity. Although the CHD-specific content addressed emotional responses to living with the condition, it may not have been sustained enough to influence deeply held identity constructs.^60^ Prior research suggests identity processes are relatively stable and may require longer-term, reflective approaches such as narrative therapy.^71^

From an implementation perspective, the digital format offers practical advantages.^39,70,72^ Many adults with CHD face barriers to accessing psychological care, including a lack of specialized clinicians, time constraints, and geographic limitations.^35,36^ The current intervention presents a low-burden solution that can be delivered alongside standard cardiac care, allowing broad adoption.^6,7,8,9^

### Strengths and Limitations

This study has several strengths, including a large and diagnostically confirmed sample, a rigorously controlled trial design, and a theory-based, contextually tailored intervention. However, several limitations warrant consideration. First, outcomes were assessed using validated self-report instruments, which may be subject to reporting bias; future studies should incorporate clinician-rated measures and passive digital biomarkers to mitigate this risk. Second, the rate of participant attrition, while comparable with rates in other digital health trials,^73^ was slightly higher among male participants, suggesting that future interventions may benefit from gender-sensitive engagement strategies to improve retention. Third, longer follow-up assessments are needed to determine whether observed improvements are sustained over time. Fourth, although participants were blinded to the content of the alternative program, they knew they were receiving an intervention, which may have introduced expectancy effects.

## Conclusions

In this randomized clinical trial of adults with CHD, access to digital emotion regulation interventions improved psychosocial outcomes compared with usual care. A program tailored to CHD produced earlier and broader effects, whereas a general program showed benefits at follow-up. These findings address a gap in care and support the use of online tools that cultivate flexible, context-sensitive emotion regulation. As digital health technologies continue to evolve, integrating such interventions into routine care may expand access to timely, personalized support. Future research should evaluate implementation in clinical settings and adapt these programs for other chronic conditions to inform scalable models of care.

## Notes

Supplement 1.
Trial Protocol

Supplement 2. **eTable 1.** Structure of the Digital Emotion Regulation Interventions **eTable 2**. Group-by-Time Interaction Effects on Emotion Regulation Strategies **eTable 3**. Group-by-Time Interaction Effects on Illness Identity Components **eTable 4.** Predictors of Study Attrition and Intervention Adherence **eTable 5.** Frequency of Negative Effects During the Digital Interventions **eTable 6**. Within-Group Changes Based on Multiple Imputation by Chained Equations **eTable 7**. Group-by-Time Interaction Effects Based on Multiple Imputation by Chained Equations **eTable 8.** False Discovery Rate-Adjusted P Values for Secondary Outcomes **eTable 9.** Moderator Analyses of Participant Characteristics on Changes in the Primary Outcome

Supplement 3.
Data Sharing Statement
