# Supplementary material for: Digital Emotion Regulation Interventions for Patients With Congenital Heart Disease: A Randomized Clinical Trial
Source: JAMA Netw Open. 2025 Oct 24;8(10):e2538813. doi: 10.1001/jamanetworkopen.2025.38813 (PMC12552930; doi:10.1001/jamanetworkopen.2025.38813)
Supplement: Supplement 1. — Trial Protocol [file jamanetwopen-e2538813-s001.pdf]

**Efficacy of Web-Based Emotion Regulation Interventions for Patients With Congenital Heart Disease: A Randomized Controlled Trial**

***Trial Protocol***

Pruessner, Luise[1]

Hartmann, Steffen[1]

Ehmann, Anna-Lena[1]

Barnow, Sven[1]

Bauer, Ulrike[2]

Helm, Paul[2]

[1] Department of Psychology, Heidelberg University, Heidelberg (Germany)

[2] National Register for Congenital Heart Defects, Berlin (Germany)

## 1. Background and Rationale

Congenital heart disease (CHD) affects approximately 1% of live births, and due to medical advances, most of these individuals now survive into adulthood [1]. However, many adults with CHD experience ongoing psychological distress, including elevated levels of anxiety, depression, and stress [2-6]. Despite the pronounced risk for psychopathology and the interest in psychological treatments, access to tailored psychosocial interventions remains limited [7, 8]. The COVID-19 pandemic has added additional stressors to the chronic strain of this condition [9, 10], underscoring the need for empirical research into psychological treatment options.

Emotion regulation, the capacity to modulate emotional responses, is critical for maintaining mental health [11, 12], physical well-being [13, 14], and cardiovascular health [15-17]. Interventions designed to enhance emotion regulation abilities have demonstrated efficacy across clinical and nonclinical populations, yet none have been specifically adapted for adults with CHD [18-23]. Given the unique emotional challenges posed by living with a congenital cardiac condition, there is a compelling rationale for developing and testing disease-specific emotion regulation interventions.

Digital cognitive-behavioral therapy platforms offer scalable, low-threshold delivery and have shown promise in general populations [24, 25]. Therefore, the present study investigates the efficacy of two such interventions: one specifically tailored to the CHD experience and another offering general emotion regulation training. This trial also includes a control group with delayed access to the intervention, receiving standard care.

## 2. Objectives and Hypotheses

The study's primary objective is to evaluate the efficacy of a CHD-specific digital emotion regulation intervention in reducing emotion regulation difficulties. The secondary

objectives include assessing changes in emotion regulation strategy use, psychological well-being, life satisfaction, depressive symptoms, anxiety, perceived stress, and illness identity.

It is hypothesized that both active intervention groups (CHD-specific and general emotion regulation) will show greater improvements across all outcomes compared to the control group at post-treatment (week 4) and follow-up (week 8). Furthermore, the CHD-specific intervention is expected to yield superior outcomes relative to the general intervention due to its disease-relevant content and contextual relevance.

## 3. Study Design

This study employs a randomized, controlled, parallel-group design with three arms: (1) a CHD-specific digital emotion regulation intervention, (2) a general digital emotion regulation intervention, and (3) a control group receiving usual care. Participants are randomly assigned in a 1:1:1 ratio, and assessments are conducted at three points: baseline (week 0), post-intervention (week 4), and follow-up (week 8). Randomization is executed using a computer-generated permuted block algorithm, with allocation concealment maintained through a secure online platform [26]. Outcome assessors and data analysts remain blinded to group assignments, ensuring unbiased analysis.

### *3.1. Inclusion Criteria*

Participants will be recruited via the German National Register for Congenital Heart Defects, a centralized registry that ensures access to a representative sample of adults living with CHD in Germany [27]. To be eligible for enrollment in the trial, individuals must meet the following criteria (see Table 1): they must be at least 18 years of age, possess a confirmed medical diagnosis of congenital heart disease, and have adequate proficiency in the German language, defined as a minimum C1 level under the Common European Framework of Reference for Languages. In addition, participants must have continuous access to the

internet throughout the study period to ensure full engagement with the web-based intervention platform.

### *3.2. Exclusion Criteria*

Individuals will be excluded from participation if they do not meet one or more inclusion criteria. Further exclusion criteria include the presence of acute suicidality, which may indicate a need for a higher level of clinical care than this digital intervention can provide [28], and any condition that impairs an individual's capacity to provide informed consent. These exclusions are intended to safeguard participant well-being and ensure ethical participation in the study.

**Table 1.** *Inclusion and Exclusion Criteria*

| Inclusion Criteria | Exclusion Criteria |
|---|---|
| 1) Sufficient German language proficiency (C1) | 1) Inability to provide informed consent |
| 2) Permanent internet access during the study | 2) Acute suicidality |
| 3) Diagnosis of congenital heart disease | |
| 4) Age ≥ 18 years | |

## 4. Trial Arms

### *4.1. CHD-Specific Digital Emotion Regulation Intervention*

This four-week self-guided intervention is tailored to the emotional challenges experienced by individuals with CHD. Content includes animated psychoeducational videos, interactive exercises, and app-based ecological momentary interventions (EMIs). Topics include challenges of CHD, emotional awareness, emotion regulation, positive behavioral engagement, reappraisal, rumination, acceptance, problem-solving, avoidance reduction,

emotional expression, and flexible strategy deployment. All examples are based on CHD-specific stressors such as procedural anxiety and physical limitations (see Table 2).

### *4.2. General Digital Emotion Regulation Intervention*

The structure and delivery match the tailored intervention but utilize generic examples unrelated to CHD. Topics and strategies mirror the CHD-specific program but lack contextual tailoring.

### *4.3. Ecological Momentary Intervention Component*

Both interventions include EMIs to promote real-time emotion regulation practice. Users receive random prompts to identify their emotions and apply strategies accordingly. This component operationalizes the extended process model of emotion regulation [29, 30].

### *4.4. Control Group*

Participants in the control group received no intervention during the study period but continued with standard CHD care. After the final assessment, they gain access to both digital interventions.

**Table 2.** *Trial Arms and Interventions*

| Group | Description | Delivery Mode |
|---|---|---|
| CHD-specific intervention | 4-week digital emotion regulation program tailored to CHD-specific emotional challenges | Usual care, immediate access to the web-based platform |
| General intervention | 4-week digital emotion regulation program identical in structure but not tailored to CHD | Usual care, immediate access to the web-based platform |
| Control group | No intervention during the study; post-trial access to both interventions | Usual care, post-trial access to the web-based platforms |

## 5. Outcome Measures

### *5.1. Primary Outcome*

The primary outcome of this trial is the level of emotion regulation difficulties, assessed using the *Difficulties in Emotion Regulation Scale* [DERS; 31]. The DERS is a widely validated, 36-item self-report instrument that evaluates multiple dimensions of impaired emotion regulation, including nonacceptance of emotional responses, difficulties engaging in goal-directed behavior, impulse control difficulties, lack of emotional awareness, limited access to emotion regulation strategies, and lack of emotional clarity. Participants rate each item on a 5-point Likert scale ranging from 1 ('almost never') to 5 ('almost always'), with higher scores indicating greater emotion dysregulation. The DERS has demonstrated excellent psychometric properties in clinical and nonclinical populations, including German-language samples.

### *5.2. Secondary Outcomes*

A range of secondary outcomes will be assessed to capture broader psychosocial functioning and emotional well-being:

- <u>Emotion Regulation Strategy Repertoire:</u> Assessed using the *Heidelberg Form for Emotion Regulation Strategies* [HFERST; 32], a validated self-report questionnaire measuring the frequency and flexibility of emotion regulation strategy use. It covers eight emotion regulation strategies across intrapersonal and interpersonal domains.
- <u>Psychological Well-Being:</u> Measured with the *World Health Organization-Five Well-Being Index* [WHO-5; 33], a brief, 5-item scale assessing subjective psychological well-being over the previous two weeks. Items are rated on a 6-point Likert scale, with higher

scores reflecting greater well-being. The WHO-5 is widely used and has shown robust reliability and validity across diverse populations, including German speakers.

- Life Satisfaction: Assessed via the *Satisfaction with Life Scale* [SWLS; 34], a 5-item instrument that evaluates global cognitive judgments of life satisfaction. Items are rated on a 7-point scale from “strongly disagree” to “strongly agree.”
- Depressive Symptoms: Evaluated using the *Patient Health Questionnaire*-9 [PHQ-9; 35], a 9-item measure aligned with DSM-5 criteria for major depressive disorder. It assesses the frequency of depressive symptoms over the past two weeks, using a 4-point scale from “not at all” to “nearly every day.” The PHQ-9 has demonstrated excellent internal consistency and diagnostic accuracy in clinical and general populations.
- Anxiety Symptoms: Measured using the *Generalized Anxiety Disorder Scale-7* [GAD-7; 36], a 7-item instrument assessing the severity of generalized anxiety symptoms. Like the PHQ-9, it uses a 4-point frequency scale and is validated for screening and severity monitoring.
- Perceived Stress: Assessed using the *Perceived Stress Scale* [PSS; 37], capturing perceived stress and coping inefficacy over the past month. Items reflect unpredictability, uncontrollability, and overload in life situations, rated on a 5-point scale.
- Illness Identity: Measured by the *Illness Identity Questionnaire* [IIQ; 38], which assesses how individuals cognitively and emotionally integrate their illness into their sense of self. The IIQ distinguishes among four identity states: engulfment, rejection, acceptance, and enrichment. It is particularly relevant for chronic conditions such as CHD.

**Table 3.** *SPIRIT schedule of the randomized controlled trial*

| | STUDY PERIOD | | | | |
|---|---|---|---|---|---|
| | Enrollment | Allocation | Baseline | Post | Follow-up |
| **ENROLLMENT** | | | | | |
| Eligibility screen | + | | | | |
| Informed consent | + | | | | |
| **ALLOCATION** | | | | | |
| Randomization | | + | | | |
| **INTERVENTIONS** | | | | | |
| CHD-specific digital intervention | | + | + | | |
| General digital intervention | | + | + | | |
| Control group with access to usual care | | + | | | |
| **ASSESSMENTS** | | | | | |
| Emotion regulation [DERS; 31] | | | + | + | + |
| Emotion strategies [HFERST; 32] | | | + | + | + |
| Well-being [WHO-5; 33] | | | + | + | + |
| Life satisfaction [SWLS; 34] | | | + | + | + |
| Depression [PHQ-9; 35] | | | + | + | + |
| Anxiety [GAD-7; 36] | | | + | + | + |
| Perceived stress [PSS; 37] | | | + | + | + |
| Illness identity [IIQ; 38] | | | + | + | + |

## 6. Statistical Analysis Plan

Data will be analyzed using intention-to-treat principles. Linear mixed-effects models will be employed to assess changes over time and between groups, with fixed effects for group, time, and group-by-time interactions and random intercepts for participants. Cohen's *d* will be calculated using pooled baseline standard deviations [39].

Missing data will be addressed through multiple imputations via chained equations (MICE). All statistical tests will be two-tailed with significance set at $p < .05$. Bonferroni-Holm corrections will be applied to account for multiple comparisons.

Moderator analyses will explore whether baseline characteristics (e.g., age, sex, current treatment, CHD severity, baseline anxiety, depression, stress, well-being, and life satisfaction) influence treatment outcomes (see Table 4).

**Table 4.** *Statistical Analysis Plan Overview*

| Analysis | Method | Missing Data | Multiplicity |
|---|---|---|---|
| 1) Outcome analyses | Mixed-effects models | Mixed-effects models | Bonferroni-Holm |
| 2) Effect size estimation | Cohen's *d* | Multiple imputation | |
| 3) Moderation analyses | Moderator × time × group | | |

## 7. Ethical Considerations and Dissemination

The ethics committees at Heidelberg University (AZ Prüß 2022 1/1) and Charité-Universitätsmedizin Berlin (EA2/108/21) have approved the study protocol. All participants provide informed consent before enrollment. Data are collected and stored in compliance with the European General Data Protection Regulation standards. Safety monitoring includes regular review of participant-reported outcomes and EMI responses to detect signs of acute distress. Results will be disseminated through peer-reviewed publications and presentations at

national and international conferences. Trial outcomes will also be registered and publicly available via ClinicalTrials.gov (https://clinicaltrials.gov).

## 8. Discussion

With a prevalence of about 1%, congenital heart disease is the most common congenital condition in humans [1]. Due to significant medical advances, survival rates have improved, with approximately 90% reaching adulthood [40]. With adulthood, psychosocial factors and mental health become increasingly relevant alongside medical care [41]. CHD patients have a higher prevalence of psychological disorders compared to the general population [2-6]. Despite evidence of an elevated risk for psychopathology and interest among patients in psychological treatments, research on interventions for CHD-related psychological distress remains scarce [7, 8]. Few specific intervention studies exist in the context of CHD; one preliminary study [42] on stress management and resilience enhancement reported feasibility but could not confirm effectiveness due to a small sample size.

Emotion regulation interventions present a promising avenue for fostering adjustment to illness-related and emotional burdens [24, 25]. Research during the COVID-19 pandemic has shown that successful emotion regulation correlates with resilience, while difficulties in emotional management predict increased anxiety, depression, and stress [13, 43-49]. The online emotion regulation intervention thus offers a flexible, accessible solution for promoting adaptive coping with illness-related emotional burdens, benefiting CHD patients both during the pandemic and beyond [24, 25]. Given the specific medical and emotional stressors faced by CHD patients [7, 41], evaluating disease-specific programs beyond general emotion regulation interventions is warranted.

The present trial seeks to address these critical gaps in the literature by evaluating a novel, CHD-specific digital intervention designed to improve emotion regulation in adults

living with CHD. Unlike general programs, this intervention was developed with direct reference to the unique psychosocial demands experienced by this population. It incorporates tailored psychoeducational content, interactive cognitive-behavioral strategies, and real-time ecological momentary interventions (EMIs) that reflect the lived experience of CHD, such as anticipatory anxiety before medical procedures, coping with physical limitations, and navigating uncertainty about one's health trajectory. By integrating contextualized content with evidence-based emotion regulation strategies, the intervention seeks to reduce emotional distress and enhance participants' sense of agency in managing their emotional responses to illness-related stressors [2-6].

Notably, the trial also includes a structurally equivalent general emotion regulation program as an active comparator [18-23], enabling a rigorous evaluation of the added value of disease-specific tailoring. Both interventions are matched in format, duration, delivery platform, and core strategies, thereby isolating the effects of contextual relevance. This comparison addresses a key question in digital intervention design, namely, whether tailoring content to the specific experiences of a clinical population results in greater psychological benefit than generalized programs. Including a third arm, a control group receiving usual care, further strengthens the study design by providing a baseline comparison against standard care practices, which currently lack structured psychosocial support. The triadic structure of this randomized trial allows for the differentiation of specific, nonspecific, and contextual effects, contributing valuable insights to the broader literature on digital mental health interventions [50-52].

Outcome assessment spans a broad range of clinically and theoretically relevant domains, including proximal mechanisms (emotion regulation skills and strategy repertoire) and distal outcomes (psychological well-being, life satisfaction, depressive and anxiety symptoms, perceived stress, and illness identity). This comprehensive outcome framework

supports the investigation of both efficacy and mechanisms of change. Furthermore, using standardized, psychometrically validated instruments ensures the reliability and interpretability of findings [31]. Assessments at baseline, post-intervention, and follow-up allow immediate and short-term sustained effects to be evaluated, which is essential in determining the intervention's real-world utility. Including moderator analyses will further elucidate for whom and under what conditions the intervention is most effective, potentially guiding the development of more personalized intervention strategies.

Finally, recruitment through the German National Register for Congenital Heart Defects offers a rare opportunity to engage a representative and well-characterized population of adults with CHD across a broad clinical and demographic spectrum [27]. This enhances the ecological validity and generalizability of the study findings. Given the persistent barriers to accessing mental health services, especially among medically vulnerable or geographically dispersed populations, the accessibility and scalability of this digital intervention represent important strengths [50-52]. Should the CHD-specific intervention prove effective, it could be readily implemented as part of routine psychosocial care in cardiology clinics, patient organizations, or national care registries. Moreover, the trial's design and methodological rigor may serve as a model for future digital mental health interventions targeting other populations with chronic or congenital medical conditions who face similarly under-addressed emotional health needs.

## References

1. Tennant, P.W., M.S. Pearce, M. Bythell, and J. Rankin, *20-year survival of children born with congenital anomalies: a population-based study.* The Lancet, 2010. **375**(9715): p. 649-56.

2. Jackson, J.L., C.E. Leslie, and S.N. Hondorp, *Depressive and Anxiety Symptoms in Adult Congenital Heart Disease: Prevalence, Health Impact and Treatment.* Progress in Cardiovascular Diseases, 2018. **61**(3-4): p. 294-299.

3. Pauliks, L.B., *Depression in adults with congenital heart disease-public health challenge in a rapidly expanding new patient population.* World Journal of Cardiology, 2013. **5**(6): p. 186-95.

4. Kessler, R.C. and E.J. Bromet, *The epidemiology of depression across cultures.* Annual Review of Public Health, 2013. **34**: p. 119-38.

5. Bandelow, B. and S. Michaelis, *Epidemiology of anxiety disorders in the 21st century.* Dialogues in Clinical Neuroscience, 2015. **17**(3): p. 327-35.

6. Westhoff-Bleck, M., J. Briest, D. Fraccarollo, D. Hilfiker-Kleiner, L. Winter, U. Maske, M.A. Busch, S. Bleich, J. Bauersachs, and K.G. Kahl, *Mental disorders in adults with congenital heart disease: Unmet needs and impact on quality of life.* Journal of Affective Disorders, 2016. **204**: p. 180-6.

7. Andonian, C., J.r. Beckmann, S. Biber, P. Ewert, S. Freilinger, H. Kaemmerer, R. Oberhoffer, L. Pieper, and R.C. Neidenbach, *Current research status on the psychological situation of adults with congenital heart disease.* Cardiovascular Diagnosis and Therapy, 2018. **8**(6): p. 799-804.

8. Kovacs, A., K.L. Bendell, J. Colman, J.L. Harrison, E. Oechslin, and C. Silversides, *Adults with congenital heart disease: psychological needs and treatment preferences.* Congenital Heart Disease, 2009. **4**(3): p. 139-46.

9. Radke, R.M., T. Frenzel, H. Baumgartner, and G.-P. Diller, *Adult congenital heart disease and the COVID-19 pandemic.* Heart, 2020. **106**(17): p. 1302-1309.

10. Diller, G.-P., M.A. Gatzoulis, C.S. Broberg, J. Aboulhosn, M. Brida, M. Schwerzmann, M. Chessa, A.H. Kovacs, and J. Roos-Hesselink, *Coronavirus disease 2019 in adults with congenital heart disease: a position paper from the ESC working group of adult congenital heart disease, and the International Society for Adult Congenital Heart Disease.* European heart journal, 2020: p. ehaa960.

11. Cludius, B., D. Mennin, and T. Ehring, *Emotion regulation as a transdiagnostic process.* Emotion, 2020. **20**(1): p. 37-42.

12. Berking, M. and P. Wupperman, *Emotion regulation and mental health: recent findings, current challenges, and future directions.* Current opinion in psychiatry, 2012. **25**(2): p. 128-134.

13. Cloitre, M., C. Khan, M.-A. Mackintosh, D.W. Garvert, C.M. Henn-Haase, E.C. Falvey, and J. Saito, *Emotion regulation mediates the relationship between ACES and physical and mental health.* Psychological Trauma: Theory, Research, Practice, and Policy, 2019. **11**(1): p. 82.

14. Kokkonen, M., *Emotion regulation and physical health in adulthood: A longitudinal, personality-oriented approach.* 2003.

15. Appleton, A.A. and L.D. Kubzansky, *Emotion regulation and cardiovascular disease risk*, J.J. Gross, Editor. 2014, The Guilford Press. p. 596–612.

16. Appleton, A.A., E.B. Loucks, S.L. Buka, and L.D. Kubzansky, *Divergent associations of antecedent-and response-focused emotion regulation strategies with midlife cardiovascular disease risk.* Annals of Behavioral Medicine, 2014. **48**(2): p. 246-255.

17. Roy, B., C. Riley, and R. Sinha, *Emotion regulation moderates the association between chronic stress and cardiovascular disease risk in humans: a cross-sectional study.* Stress, 2018. **21**(6): p. 548-555.

18. Berking, M., *Training Emotionaler Kompetenzen*. 2017, Berlin Heidelberg: Springer.

19. Schuppert, H.M., M.E. Timmerman, J. Bloo, T.G. van Gemert, H.M. Wiersema, R.B. Minderaa, P.M. Emmelkamp, and M.H. Nauta, *Emotion regulation training for adolescents with borderline personality disorder traits: A randomized controlled trial.* Journal of the American Academy of Child & Adolescent Psychiatry, 2012. **51**(12): p. 1314-1323.

20. Barnow, S., E. Reinelt, and C. Sauer, *Emotionsregulation: Manual und Materialien für Trainer und Therapeuten (Psychotherapie: Praxis)* 2016: Springer.

21. Bahrami, S., M. Sheikhi, M. Moradi Baglooei, and M. Mafi, *Effect of Emotion Regulation Training Based on the Gross Model on Anxiety among Parents of Children with Cancer.* Evidence Based Care, 2020. **9**(4): p. 40-47.

22. Behrouian, M., T. Ramezani, M. Dehghan, A. Sabahi, and B.E. Zarandi, *The effect of emotion regulation training on stress, anxiety, and depression in family caregivers of patients with schizophrenia: a randomized controlled trial.* Community Mental Health Journal, 2020: p. 1-8.

23. Saedpanah, D., S. Salehi, and L.F. Moghaddam, *The effect of emotion regulation training on occupational stress of critical care nurses.* Journal of Clinical and Diagnostic Research, 2016. **10**(12): p. VC01–VC04.

24. Goldin, P., M. Ziv, H. Jazaieri, K. Werner, H. Kraemer, R. Heimberg, and J. Gross, *Cognitive Reappraisal Self-Efficacy Mediates the Effects of Individual Cognitive-Behavioral Therapy for Social Anxiety Disorder.* Journal of consulting and clinical psychology, 2012. **80**.

25. Kivity, Y. and J. Huppert, *Does Cognitive Reappraisal Reduce Anxiety? A Daily Diary Study of a Micro-Intervention With Individuals With High Social Anxiety.* Journal of Consulting and Clinical Psychology, 2016. **84**.

26. Leiner, D.J., *SoSci Survey*. 2021.

27. Helm, P.C., M.A. Koerten, H. Abdul-Khaliq, H. Baumgartner, D. Kececioglu, and U.M. Bauer, *Representativeness of the German National Register for Congenital Heart Defects: a clinically oriented analysis.* Cardiology in the Young, 2016. **26**(5): p. 921-6.

28. Sander, L., K. Gerhardinger, E. Bailey, J. Robinson, J. Lin, P. Cuijpers, and C. Mühlmann, *Suicide risk management in research on internet-based interventions for depression: A synthesis of the current state and recommendations for future research.* Journal of Affective Disorders, 2020. **263**: p. 676-683.

29. Gross, J.J., *Emotion Regulation: Current Status and Future Prospects.* Psychological Inquiry, 2015. **26**(1): p. 1-26.

30. McRae, K. and J.J. Gross, *Emotion regulation.* Emotion, 2020. **20**(1): p. 1-9.

31. Gratz, K.L. and L. Roemer, *Multidimensional assessment of emotion regulation and dysregulation: Development, factor structure, and initial validation of the difficulties in emotion regulation scale.* Journal of Psychopathology and Behavioral Assessment, 2004. **26**(1): p. 41-54.

32. Izadpanah, S., S. Barnow, A.B. Neubauer, and J. Holl, *Development and validation of the Heidelberg Form for Emotion Regulation Strategies (HFERST): Factor structure, reliability, and validity.* Assessment, 2019. **26**(5): p. 880-906.

33. Bech, P., L.R. Olsen, M. Kjoller, and N.K. Rasmussen, *Measuring well-being rather than the absence of distress symptoms: a comparison of the SF-36 Mental Health subscale and the WHO-Five well-being scale.* International Journal of Methods in Psychiatric Research, 2003. **12**(2): p. 85-91.

34. Glaesmer, H., G. Grande, E. Braehler, and M. Roth, *The German version of the satisfaction with life scale (SWLS): Psychometric properties, validity, and population-based norms.* European Journal of Psychological Assessment, 2011. **27**(2): p. 127-132.

35. Kroenke, K., R.L. Spitzer, and J.B. Williams, *The PHQ-9: validity of a brief depression severity measure.* Journal of General Internal Medicine, 2001. **16**(9): p. 606-613.

36. Löwe, B., O. Decker, S. Müller, E. Brähler, D. Schellberg, W. Herzog, and P.Y. Herzberg, *Validation and standardization of the Generalized Anxiety Disorder Screener (GAD-7) in the general population.* Medical Care, 2008: p. 266-274.

37. Reis, D., D. Lehr, E. Heber, and D.D. Ebert, *The German version of the Perceived Stress Scale (PSS-10): evaluation of dimensionality, validity, and measurement invariance with exploratory and confirmatory bifactor modeling.* Assessment, 2019. **26**(7): p. 1246-1259.

38. Oris, L., K. Luyckx, J. Rassart, L. Goubert, E. Goossens, S. Apers, S. Arat, J. Vandenberghe, R. Westhovens, and P. Moons, *Illness Identity in Adults with a Chronic Illness.* Journal of Clinical Psychology in Medical Settings, 2018. **25**: p. 429-440.

39. Morris, S.B. and R.P. DeShon, *Combining effect size estimates in meta-analysis with repeated measures and independent-groups designs.* Psychological Methods, 2002. **7**(1): p. 105.

40. Diller, G.-P., G. Breithardt, and H. Baumgartner, *Congenital heart defects in adulthood.* Deutsches Arzteblatt international, 2011. **108**(26): p. 452-459.

41. Kovacs, A.H., S.F. Sears, and A.S. Saidi, *Biopsychosocial experiences of adults with congenital heart disease: Review of the literature.* American Heart Journal, 2005. **150**(2): p. 193-201.

42. Kovacs, A.H., S.L. Grace, A.C. Kentner, R.P. Nolan, C.K. Silversides, and M.J. Irvine, *Feasibility and Outcomes in a Pilot Randomized Controlled Trial of a Psychosocial Intervention for Adults With Congenital Heart Disease.* Canadian Journal of Cardiology, 2018. **34**(6): p. 766-773.

43. Denny, B.T. and K.N. Ochsner, *Behavioral effects of longitudinal training in cognitive reappraisal.* Emotion, 2014. **14**(2): p. 425-33.

44. Gross, J.J., *Antecedent- and response-focused emotion regulation: Divergent consequences for experience, expression, and physiology.* Journal of personality and social psychology, 1998. **74**(1): p. 224-237.

45. Jackson, D.C., J.R. Malmstadt, C.L. Larson, and R.J. Davidson, *Suppression and enhancement of emotional responses to unpleasant pictures.* Psychophysiology, 2000. **37**(4): p. 515-22.

46. Veer, I., A. Riepenhausen, M. Zerban, C. Wackerhagen, H. Engen, L. Puhlmann, G. Köber, S. Bögemann, J. Weermeijer, A. Uściłko, N. Mor, G. Barsuola, P. Cardone, Y. Deza-Araujo, K. Farkas, C. Feller, M. Hajdúk, L. Ilen, Z. Kasanova, and R. Kalisch, *Mental resilience in the Corona lockdown: First empirical insights from Europe*. 2020.

47. Brehl, A.-K., A. Schene, N. Kohn, and G. Fernández, *Maladaptive emotion regulation strategies in a vulnerable population predict increased anxiety during the Covid-19 pandemic: a pseudo-prospective study.* Journal of Affective Disorders Reports, 2021. **4**: p. 100113.

48. Gubler, D.A., L.M. Makowski, S.J. Troche, and K. Schlegel, *Loneliness and well-being during the Covid-19 pandemic: Associations with personality and emotion regulation.* Journal of Happiness Studies, 2020: p. 1-20.

49. Panayiotou, G., M. Panteli, and C. Leonidou, *Coping with the invisible enemy: The role of emotion regulation and awareness in quality of life during the COVID-19 pandemic.* Journal of Contextual Behavioral Science, 2021. **19**: p. 17-27.

50. Kaihara, T., M. Scherrenberg, V. Intan-Goey, M. Falter, H. Kindermans, I. Frederix, and P. Dendale, *Efficacy of digital health interventions on depression and anxiety in patients with cardiac disease: a systematic review and meta-analysis.* European Heart Journal-Digital Health, 2022. **3**(3): p. 445-454.

51. Whitelaw, S., D.M. Pellegrini, and M.A. Mamas, *Barriers and facilitators of the uptake of digital health technology in cardiovascular care: a systematic scoping review.* European Heart Journal - Acute Cardiovascular Care, 2021. **2**(1): p. 62-69.

52. Rudd, B.N. and R.S. Beidas, *Digital mental health: the answer to the global mental health crisis?* JMIR Mental Health, 2020. **7**(6): p. e18472.
