# Supplementary material for: Digital Emotion Regulation Interventions for Patients With Congenital Heart Disease: A Randomized Clinical Trial
Source: JAMA Netw Open. 2025 Oct 24;8(10):e2538813. doi: 10.1001/jamanetworkopen.2025.38813 (PMC12552930; doi:10.1001/jamanetworkopen.2025.38813)
Supplement: Supplement 2. — eTable 1. Structure of the Digital Emotion Regulation Interventions eTable 2. Group-by-Time Interaction Effects on Emotion Regulation Strategies eTable 3. Group-by-Time Interaction Effects on Illness Identity Components eTable 4. Predictors of Study Attrition and Intervention Adherence eTable 5. Frequency of Negative Effects During the Digital Interventions eTable 6. Within-Group Changes Based on Multiple Imputation by Chained Equations eTable 7. Group-by-Time Interaction Effects Based on Multiple Imputation by Chained Equations eTable 8. False Discovery Rate-Adjusted P Values for Secondary Outcomes eTable 9. Moderator Analyses of Participant Characteristics on Changes in the Primary Outcome [file jamanetwopen-e2538813-s002.pdf]

Supplementary Online Content

Pruessner L, Hartmann S, Ehmann AL, Barnow S, Bauer UMM, Helm PC. Digital emotion regulation interventions for patients with congenital heart disease: a randomized clinical trial. *JAMA Netw Open.* 2025;8(10):e2538813. doi:10.1001/jamanetworkopen.2025.38813

eTable 1. Structure of the Digital Emotion Regulation Interventions
eTable 2. Group-by-Time Interaction Effects on Emotion Regulation Strategies
eTable 3. Group-by-Time Interaction Effects on Illness Identity Components
eTable 4. Predictors of Study Attrition and Intervention Adherence
eTable 5. Frequency of Negative Effects During the Digital Interventions
eTable 6. Within-Group Changes Based on Multiple Imputation by Chained Equations
eTable 7. Group-by-Time Interaction Effects Based on Multiple Imputation by Chained Equations
eTable 8. False Discovery Rate-Adjusted P Values for Secondary Outcomes
eTable 9. Moderator Analyses of Participant Characteristics on Changes in the Primary Outcome

This supplementary material has been provided by the authors to give readers additional information about their work.

© 2025 Pruessner L et al. *JAMA Network Open.*

eTable 1. Structure of the Digital Emotion Regulation Interventions

Lesson	General Emotion Regulation Intervention			Tailored Emotion Regulation Intervention		
	Topic	Format	Activity	Topic	Format	Activity
1	Identifying Emotions	Video	Psychoeducation	Challenges of CHD	Exercise	Psychoeducation
2	Emotion Awareness	Exercise	Ecological Momentary Intervention	Identifying Emotions	Video	Psychoeducation
3	Purpose of Emotions	Exercise	Ecological Momentary Intervention	Emotion Awareness	Exercise	Ecological Momentary Intervention
4	Emotion Regulation	Video	Psychoeducation	Purpose of Emotions	Exercise	Ecological Momentary Intervention
5	Goal Setting	Exercise	Ecological Momentary Intervention	Emotion Regulation	Video	Psychoeducation
6	Positive Behavioral Engagement	Video	Psychoeducation	Goal Setting	Exercise	Ecological Momentary Intervention
7	Mixed Emotions	Exercise	Ecological Momentary Intervention	Positive Behavioral Engagement	Video	Psychoeducation
8	Cognitive Reappraisal	Video	Psychoeducation	Mixed Emotions	Exercise	Ecological Momentary Intervention
9	Reappraisal Tactics	Exercise	Ecological Momentary Intervention	Cognitive Reappraisal	Video	Psychoeducation
10	Finding Positive Reappraisals	Exercise	Ecological Momentary Intervention	Reappraisal Tactics	Exercise	Ecological Momentary Intervention
11	Recognizing Rumination	Video	Psychoeducation	Finding Positive Reappraisals	Exercise	Ecological Momentary Intervention
12	Reducing Rumination	Exercise	Ecological Momentary Intervention	Recognizing Rumination	Video	Psychoeducation
13	Regulation Practice I	Audio	Ecological Momentary Intervention	Reducing Rumination	Exercise	Ecological Momentary Intervention
14	Consolidating Behavioral Engagement	Exercise	Ecological Momentary Intervention	Regulation Practice I	Audio	Ecological Momentary Intervention
15	Relaxation and Recovery	Exercise	Relaxation Techniques	Consolidating Behavioral Engagement	Exercise	Ecological Momentary Intervention
16	Accepting Emotions	Video	Psychoeducation	Accepting Emotions	Video	Psychoeducation

© 2025 Pruessner L et al. *JAMA Network Open.*

Lesson	General Emotion Regulation Intervention			Tailored Emotion Regulation Intervention		
	Topic	Format	Activity	Topic	Format	Activity
17	Accepting Emotions	Audio	Ecological Momentary Intervention	Accepting Emotions	Audio	Ecological Momentary Intervention
18	Regulation Practice II	Exercise	Ecological Momentary Intervention	Regulation Practice II	Exercise	Ecological Momentary Intervention
19	Problem Solving	Video	Psychoeducation	Problem Solving	Video	Psychoeducation
20	Acceptance vs Situation Modification	Exercise	Ecological Momentary Intervention	Acceptance vs Situation Modification	Exercise	Ecological Momentary Intervention
21	Regulation Practice III	Exercise	Ecological Momentary Intervention	Regulation Practice III	Exercise	Ecological Momentary Intervention
22	Reducing Avoidance	Video	Psychoeducation	Reducing Avoidance	Video	Psychoeducation
23	Regulatory Self-Efficacy	Exercise	Ecological Momentary Intervention	Regulatory Self-Efficacy	Exercise	Ecological Momentary Intervention
24	Regulation Practice IV	Exercise	Ecological Momentary Intervention	Regulation Practice IV	Exercise	Ecological Momentary Intervention
25	Expression vs Suppression	Video	Psychoeducation	Expression vs Suppression	Video	Psychoeducation
26	Writing About Emotions	Writing	Ecological Momentary Intervention	Writing About Emotions	Writing	Ecological Momentary Intervention
27	Regulation Practice V	Exercise	Ecological Momentary Intervention	Regulation Practice V	Exercise	Ecological Momentary Intervention
28	Monitoring Strategy Use	Video	Psychoeducation	Monitoring Strategy Use	Video	Psychoeducation

Abbreviations: CHD, congenital heart disease. The intervention pairs brief psychoeducational video lessons with in-the-moment ecological momentary intervention exercises. The general intervention uses standard, non-disease-specific examples, whereas the CHD-tailored intervention embeds scenarios (eg, procedural anxiety, activity limitations) relevant to congenital heart disease. Modules progress from emotion identification to strategy monitoring over 4 weeks, following the Extended Process Model of Emotion Regulation (Gross, 2015).

© 2025 Pruessner L et al. *JAMA Network Open.*

eTable 2. Group-by-Time Interaction Effects on Emotion Regulation Strategies

Outcome		Postintervention effects		Follow-up effects	
	Comparison	*b* (*SE*)	*P* value	*b* (*SE*)	*P* value
Reappraisal [HFERST]	Tailored vs usual care	0.21 (0.05)	<.001	0.21 (0.05)	<.001
	General vs usual care	0.03 (0.05)	.48	0.11 (0.05)	.02
	Tailored vs general	0.35 (0.12)	.004	0.23 (0.12)	.06
Acceptance [HFERST]	Tailored vs usual care	0.18 (0.05)	<.001	0.20 (0.05)	<.001
	General vs usual care	0.07 (0.05)	.18	0.10 (0.05)	.06
	Tailored vs general	0.23 (0.12)	.05	0.20 (0.12)	.10
Problem solving [HFERST]	Tailored vs usual care	0.06 (0.04)	.16	0.11 (0.04)	.006
	General vs usual care	0.01 (0.04)	.85	-0.06 (0.04)	.12
	Tailored vs general	0.10 (0.09)	.28	0.34 (0.09)	<.001
Social support [HFERST]	Tailored vs usual care	0.08 (0.05)	.11	0.08 (0.05)	.14
	General vs usual care	0.06 (0.05)	.28	0.01 (0.06)	.83
	Tailored vs general	0.05 (0.12)	.67	0.18 (0.12)	.15
Rumination [HFERST]	Tailored vs usual care	-0.09 (0.05)	.05	-0.12 (0.05)	.02
	General vs usual care	-0.06 (0.05)	.21	-0.09 (0.05)	.06
	Tailored vs general	-0.06 (0.11)	.55	-0.04 (0.11)	.70
Expressive suppression [HFERST]	Tailored vs usual care	-0.07 (0.04)	.11	-0.04 (0.04)	.31
	General vs usual care	-0.04 (0.04)	.41	-0.09 (0.04)	.04
	Tailored vs general	-0.07 (0.10)	.47	0.10 (0.11)	.36
Experiential suppression [HFERST]	Tailored vs usual care	-0.09 (0.04)	.02	-0.08 (0.04)	.05
	General vs usual care	-0.002 (0.04)	.97	-0.06 (0.04)	.20
	Tailored vs general	-0.17 (0.09)	.049	-0.05 (0.09)	.62
Avoidance [HFERST]	Tailored vs usual care	-0.10 (0.05)	.046	-0.11 (0.05)	.03
	General vs usual care	-0.01 (0.05)	.87	-0.12 (0.05)	.03
	Tailored vs general	-0.19 (0.12)	.11	-0.01 (0.12)	.93

Abbreviations: HFERST, Heidelberg Form for Emotion Regulation Strategies. Data are group-by-time interaction effects from linear mixed-effects models. Regression coefficients (*b*) reflect the between-group difference in change (first group minus second): negative values indicate a larger reduction over time in the first group; positive values indicate a larger increase in the first group. Because some strategies are expected to decrease (ie, rumination, suppression, avoidance) and others to increase (ie, reappraisal, acceptance, problem solving), interpret the sign accordingly. *P* values are 2-sided.

© 2025 Pruessner L et al. *JAMA Network Open.*

eTable 3. Group-by-Time Interaction Effects on Illness Identity Components

Outcome		Postintervention effects		Follow-up effects	
	Comparison	*b* (SE)	*P* value	*b* (SE)	*P* value
Enrichment [IIQ]	Tailored vs usual care	0.02 (0.05)	.64	0.05 (0.04)	.28
	General vs usual care	0.02 (0.04)	.70	0.04 (0.05)	.34
	Tailored vs general	0.005 (0.11)	.97	-0.005 (0.10)	.96
Acceptance [IIQ]	Tailored vs usual care	0.004 (0.03)	.90	0.002 (0.04)	.95
	General vs usual care	-0.01 (0.04)	.69	-0.02 (0.03)	.50
	Tailored vs general	-0.02 (0.08)	.82	-0.04 (0.08)	.61
Engulfment [IIQ]	Tailored vs usual care	0.02 (0.02)	.51	-0.01 (0.02)	.61
	General vs usual care	-0.003 (0.03)	.92	-0.04 (0.03)	.20
	Tailored vs general	0.03 (0.06)	.57	0.05 (0.06)	.43
Rejection [IIQ]	Tailored vs usual care	-0.05 (0.03)	.14	-0.03 (0.04)	.43
	General vs usual care	-0.02 (0.03)	.55	-0.01 (0.03)	.77
	Tailored vs general	0.06 (0.07)	.44	0.04 (0.08)	.65

Abbreviations: IIQ, Illness Identity Questionnaire. Data are group-by-time interaction effects from linear mixed-effects models. Regression coefficients (*b*) indicate the between-group difference in change in IIQ component scores (first group minus second): negative values indicate a larger reduction over time in the first group; positive values indicate a larger increase in the first group. Because some IIQ components are expected to decrease (ie, engulfment, rejection) and others to increase (ie, enrichment, acceptance), interpret the sign accordingly. *P* values are 2-sided.

© 2025 Pruessner L et al. *JAMA Network Open.*

eTable 4. Predictors of Study Attrition and Intervention Adherence

	Study attrition		Intervention adherence	
	OR (95% CI)	*P* value	*b* (95% CI)	*P* value
Age	1.00 (0.98 to 1.01)	.74	0.26 (-0.02 to 0.54)	.06
Gender identity	0.59 (0.40 to 0.88)	.009	2.12 (-3.36 to 7.59)	.44
Education level	0.91 (0.80 to 1.04)	.18	0.42 (-1.45 to 2.29)	.65
Psychotherapy	0.62 (0.34 to 1.13)	.11	1.78 (-4.26 to 7.83)	.56
Number of heart surgeries	1.04 (0.93 to 1.15)	.49	-0.71 (-2.27 to 0.85)	.36
Number of mental disorders	0.94 (0.77 to 1.13)	.52	-2.33 (-5.25 to 0.60)	.12
Mild CHD	0.71 (0.31 to 1.62)	.42	0.38 (-9.99 to 10.75)	.94
Moderate CHD	0.71 (0.37 to 1.42)	.32	-0.31 (-8.76 to 8.14)	.94
Severe CHD	0.70 (0.35 to 1.44)	.32	3.75 (-6.61 to 14.10)	.47

Abbreviations: CHD, congenital heart disease; CI, confidence interval; OR, odds ratio. Attrition was defined as failure to complete any postbaseline assessment; adherence was the number of completed core intervention components (video-based lessons and paired exercises), as recorded by the digital platform. ORs >1 indicate increased attrition risk; regression coefficients (*b*) represent the estimated change in completed lessons or exercises per unit increase in the predictor. *P* values are 2-sided.

© 2025 Pruessner L et al. *JAMA Network Open.*

eTable 5. Frequency of Negative Effects During the Digital Interventions

Negative effect [NEQ]	Postintervention		Follow-up	
	Tailored	General	Tailored	General
Sleep problems	0 (0)	0 (0)	0 (0)	0 (0)
Increased stress	4 (4.3)	0 (0)	0 (0)	0 (0)
Increased anxiety	0 (0)	0 (0)	0 (0)	0 (0)
Restlessness	1 (1.1)	0 (0)	0 (0)	0 (0)
Feeling down	1 (1.1)	0 (0)	0 (0)	0 (0)
Hopelessness	0 (0)	0 (0)	0 (0)	0 (0)
Low self-esteem	1 (1.1)	0 (0)	0 (0)	0 (0)
Loss of self-confidence	0 (0)	0 (0)	0 (0)	0 (0)
Sadness	1 (1.1)	0 (0)	0 (0)	0 (0)
Feeling incompetent	1 (1.1)	0 (0)	0 (0)	0 (0)
Unpleasant emotions	1 (1.1)	1 (1.1)	0 (0)	0 (0)
Issue worsened	0 (0)	0 (0)	0 (0)	0 (0)
Resurfacing memories	2 (2.2)	2 (2.2)	0 (0)	0 (0)
Lack of understanding	0 (0)	0 (0)	0 (0)	0 (0)
Lack of trust in training	0 (0)	1 (1.1)	0 (0)	0 (0)
Ineffectiveness	1 (1.1)	0 (0)	0 (0)	0 (0)
Unmet expectations	1 (1.1)	0 (0)	0 (0)	0 (0)
Training quality	1 (1.1)	0 (0)	0 (0)	0 (0)
Training not suitable	1 (1.1)	0 (0)	0 (0)	0 (0)
Demotivating experience	1 (1.1)	0 (0)	0 (0)	0 (0)
Other effects	2 (2.2)	1 (1.1)	0 (0)	0 (0)

Abbreviations: NEQ, Negative Effects Questionnaire. Values are presented as *n* (%), where *n* is the number of participants reporting the respective negative effect and % is the corresponding percentage of the sample (Tailored, *n* = 92 at postintervention, *n* = 106 at follow-up; General, *n* = 91 at postintervention, *n* = 99 at follow-up).

© 2025 Pruessner L et al. *JAMA Network Open.*

eTable 6. Within-Group Changes Based on Multiple Imputation by Chained Equations

Outcome	Group	Baseline		Postintervention change			Follow-up change		
		Intercept	SE	*b*	SE	*P* value	*b*	SE	*P* value
Difficulties in emotion regulation [DERS]	Tailored	90.33	1.80	-10.30	1.94	<.001	-13.32	2.02	<.001
	General	89.25	1.69	-6.29	1.82	<.001	-9.78	1.98	<.001
	Usual care	86.41	1.72	-2.98	1.20	.01	-4.24	1.32	<.001
Emotion regulation repertoire [HFERST]	Tailored	9.80	0.15	1.04	0.18	<.001	1.11	0.19	<.001
	General	10.29	0.14	0.33	0.18	.06	0.32	0.19	.10
	Usual care	10.01	0.14	0.13	0.10	.20	0.10	0.10	.31
Well-being [WHO-5]	Tailored	50.61	1.44	3.25	1.57	.04	3.83	1.79	.03
	General	51.54	1.59	-0.33	1.50	.82	-0.88	1.80	.63
	Usual care	52.34	1.56	-2.98	1.40	.03	-1.66	1.63	.31
Life satisfaction [SWLS]	Tailored	23.87	0.47	1.41	0.42	<.001	1.26	0.38	.001
	General	24.22	0.48	0.47	0.37	.21	0.42	0.47	.34
	Usual care	24.10	0.46	0.05	0.27	.85	0.38	0.28	.18
Anxiety [GAD-7]	Tailored	7.27	0.33	-1.28	0.40	.002	-1.44	0.42	<.001
	General	6.86	0.34	-0.66	0.38	.08	-1.03	0.38	.007
	Usual care	6.35	0.31	-0.03	0.24	.89	0.09	0.29	.75
Depression [PHQ-9]	Tailored	8.14	0.34	-1.00	0.35	.005	-0.94	0.35	.007
	General	7.79	0.35	-0.38	0.36	.29	-0.63	0.40	.12
	Usual care	7.33	0.32	-0.28	0.33	.41	-0.47	0.34	.16
Perceived stress [PSS]	Tailored	10.68	0.23	-1.10	0.28	<.001	-0.99	0.30	<.001
	General	10.68	0.23	-0.79	0.30	.008	-0.67	0.30	.02
	Usual care	10.63	0.23	0.06	0.21	.76	-0.06	0.22	.78
Illness identity [IIQ]	Tailored	3.01	0.02	0.04	0.03	.15	0.06	0.02	.02
	General	3.03	0.02	0.04	0.03	.11	0.07	0.04	.01
	Usual care	3.02	0.02	0.01	0.02	.41	0.01	0.02	.54

Abbreviations: DERS, Difficulties in Emotion Regulation Scale; WHO-5, World Health Organization Well-Being Index; SWLS, Satisfaction with Life Scale; GAD-7, Generalized Anxiety Disorder-7; PHQ-9, Patient Health Questionnaire-9; PSS, Perceived Stress Scale; IIQ, Illness Identity Questionnaire. Regression coefficients (*b*) indicate the change in the outcome score over time. *P* values are 2-sided.

© 2025 Pruessner L et al. *JAMA Network Open.*

eTable 7. Group-by-Time Interaction Effects Based on Multiple Imputation by Chained Equations

Outcome	Comparison	Postintervention effects			Follow-up effects		
		b	SE	*P* value	*b*	SE	*P* value
Difficulties in emotion regulation [DERS]	Tailored vs usual care	-3.73	1.18	.002	-4.23	1.17	.001
	General vs usual care	-1.69	1.06	.11	-3.07	1.11	.006
	Tailored vs general	-2.23	1.17	.03	-1.15	1.09	.29
Emotion regulation repertoire [HFERST]	Tailored vs usual care	0.43	0.10	<.001	0.48	0.10	<.001
	General vs usual care	0.09	0.10	.35	0.14	0.10	.19
	Tailored vs general	0.33	0.10	.002	0.35	0.10	.001
Well-being [WHO-5]	Tailored vs usual care	3.04	1.10	.006	2.79	1.23	.02
	General vs usual care	1.33	1.07	.22	0.52	1.24	.67
	Tailored vs general	1.71	1.08	.11	2.27	1.21	.06
Life satisfaction [SWLS]	Tailored vs usual care	0.67	0.28	.02	0.41	0.24	.09
	General vs usual care	0.23	0.23	.32	0.07	0.25	.78
	Tailored vs general	0.44	0.24	.07	0.34	0.26	.20
Anxiety [GAD-7]	Tailored vs usual care	-0.63	0.23	.006	-0.73	0.25	.003
	General vs usual care	-0.32	0.21	.14	-0.61	0.22	.007
	Tailored vs general	-0.31	0.22	.16	-0.12	0.24	.62
Depression [PHQ-9]	Tailored vs usual care	-0.65	0.22	.004	-0.60	0.24	.01
	General vs usual care	-0.33	0.19	.09	-0.51	0.21	.02
	Tailored vs general	-0.32	0.20	.12	-0.09	0.23	.69
Perceived stress [PSS]	Tailored vs usual care	-0.57	0.18	.002	-0.43	0.18	.02
	General vs usual care	-0.24	0.16	.13	-0.27	0.17	.10
	Tailored vs general	-0.33	0.17	.05	-0.16	0.18	.38
Illness identity [IIQ]	Tailored vs usual care	0.01	0.01	.43	0.02	0.01	.13
	General vs usual care	0.03	0.01	.06	0.03	0.01	.06
	Tailored vs general	0.02	0.01	.13	0.01	0.01	.34

Abbreviations: DERS, Difficulties in Emotion Regulation Scale; WHO-5, World Health Organization Well-Being Index; SWLS, Satisfaction with Life Scale; GAD-7, Generalized Anxiety Disorder-7; PHQ-9, Patient Health Questionnaire-9; PSS, Perceived Stress Scale; IIQ, Illness Identity Questionnaire. Regression coefficients (*b*) indicate the between-group difference in change scores (first group minus second); negative values favor greater reductions in the first-listed group. Estimates derive from the mixed-effects model and are not arithmetic differences of within-group changes. *P* values are 2-sided.

© 2025 Pruessner L et al. *JAMA Network Open.*

eTable 8. False Discovery Rate-Adjusted P Values for Secondary Outcomes

Outcome	Comparison	Postintervention effects	Follow-up effects
		P value	*P* value
Emotion regulation repertoire [HFERST]	Tailored vs usual care	<.001	<.001
	General vs usual care	.41	.17
	Tailored vs general	.04	.03
Well-being [WHO-5]	Tailored vs usual care	.008	.03
	General vs usual care	.35	.69
	Tailored vs general	.28	.33
Life satisfaction [SWLS]	Tailored vs usual care	.01	.049
	General vs usual care	.44	.73
	Tailored vs general	.28	.40
Anxiety [GAD-7]	Tailored vs usual care	.007	.007
	General vs usual care	.35	.049
	Tailored vs general	.29	.71
Depression [PHQ-9]	Tailored vs usual care	.008	.02
	General vs usual care	.35	.11
	Tailored vs general	.28	.71
Perceived stress [PSS]	Tailored vs usual care	.002	.02
	General vs usual care	.11	.11
	Tailored vs general	.40	.71
Illness identity [IIQ]	Tailored vs usual care	.41	.14
	General vs usual care	.44	.14
	Tailored vs general	1.00	1.00

Abbreviations: *P* values were adjusted for multiple comparisons using the false-discovery rate (Benjamini–Hochberg procedure). Comparisons include tailored vs usual care, general vs usual care, and tailored vs general.

© 2025 Pruessner L et al. *JAMA Network Open.*

eTable 9. Moderator Analyses of Participant Characteristics on Changes in the Primary Outcome

Moderator	Tailored vs usual care		General vs usual care		Tailored vs general	
	b (95% CI)	*P* value	*b* (95% CI)	*P* value	*b* (95% CI)	*P* value
Age	-0.14 (-0.31 to 0.02)	.09	-0.09 (-0.25 to 0.07)	.28	-0.11 (-0.51 to 0.28)	.58
Gender identity	-0.37 (-5.44 to 4.69)	.89	0.22 (-4.82 to 5.26)	.93	-0.95 (-14.60 to 11.69)	.88
Education level	0.80 (-0.72 to 2.33)	.30	0.04 (-1.51 to 1.58)	.96	1.40 (-2.28 to 5.08)	.46
Psychotherapy	-1.68 (-6.20 to 2.84)	.47	-1.01 (-5.36 to 3.34)	.65	-1.75 (-12.43 to 8.93)	.75
Number of heart surgeries	-0.71 (-1.96 to 0.53)	.27	-1.44 (-2.75 to -0.14)	.03	-1.08 (-4.24 to 2.08)	.50
Number of mental disorders	0.08 (-2.34 to 2.50)	.95	1.96 (-0.62 to 4.54)	.14	-3.22 (-8.47 to 2.04)	.23
Mild CHD	-3.25 (-14.58 to 8.08)	.57	-6.80 (-31.59 to 17.99)	.59	11.74 (-16.38 to 39.86)	.41
Moderate CHD	-3.81 (-13.71 to 6.09)	.45	-3.49 (-24.76 to 17.78)	.75	-0.80 (-24.39 to 22.80)	.95
Severe CHD	-4.85 (-15.14 to 5.45)	.36	-1.99 (-24.07 to 20.09)	.86	-2.24 (-26.54 to 22.06)	.86
Baseline life satisfaction	-0.55 (-0.93 to -0.17)	.005	-0.46 (-0.81 to -0.11)	.01	-0.18 (-1.04 to 0.69)	.69
Baseline well-being	-0.09 (-0.21 to 0.03)	.15	-0.04 (-0.14 to 0.07)	.44	-0.11 (-0.37 to 0.16)	.44
Baseline anxiety	0.69 (0.15 to 1.22)	.01	0.69 (0.19 to 1.19)	.01	0.02 (-1.12 to 1.16)	.98
Baseline depression	0.18 (-0.34 to 0.70)	.49	0.57 (0.10 to 1.04)	.02	-0.73 (-1.88 to 0.42)	.21
Baseline stress	1.35 (0.60 to 2.09)	<.001	1.14 (0.48 to 1.79)	.001	0.39 (-1.22 to 2.01)	.63

Abbreviations: CHD, congenital heart disease; CI, confidence interval; baseline life satisfaction (SWLS, Satisfaction with Life Scale); baseline well-being (WHO-5, World Health Organization Well-Being Index); baseline anxiety (GAD-7, Generalized Anxiety Disorder-7); baseline depression (PHQ-9, Patient Health Questionnaire-9); baseline stress (PSS, Perceived Stress Scale). Regression coefficients (*b*) and 95% confidence intervals (CIs) are for the time × group × moderator interaction from linear mixed-effects models with random intercepts for participants. Positive *b* indicates a larger reduction in emotion regulation difficulties over time per unit increase in the moderator; negative b indicates a larger reduction per unit decrease. *P* values are 2-sided.

.

© 2025 Pruessner L et al. *JAMA Network Open.*
