# Supplementary material for: Digital Emotion Regulation Interventions for Patients With Congenital Heart Disease: A Randomized Clinical Trial
Source: JAMA Netw Open. 2025 Oct 24;8(10):e2538813. doi: 10.1001/jamanetworkopen.2025.38813 (PMC12552930; doi:10.1001/jamanetworkopen.2025.38813)
Supplement: Supplement 3. — Data Sharing Statement [file jamanetwopen-e2538813-s003.pdf]

Data Sharing Statement

Pruessner. Digital Emotion Regulation Interventions for Patients With Congenital Heart Disease. *JAMA Netw Open*. Published October 24, 2025.
doi:10.1001/jamanetworkopen.2025.38813

Data
Additional Information: Trial Registry: ClinicalTrials.gov Trial Registration Number: NCT05862909
Data available: Yes
Data types: Deidentified participant data
How to access data: Deidentified participant data to replicate the primary analyses will be made available upon request through the Open Science Framework (https://osf.io/wyhvk).
When available: With publication

Supporting Documents
Document types: None

Additional Information
Who can access the data: The data will be made available to researchers whose proposed use of the data has been approved.
Types of analyses: Data will be accessible exclusively for research-related purposes.
Mechanisms of data availability: The data will be made available after the approval of a data access agreement. This process ensures appropriate use while facilitating accessibility.
Any additional restrictions: The supporting documents will be accessible online through the Open Science Framework (https://osf.io/wyhvk). The URL leads to a repository where all relevant documents can be requested. For any specific requests or inquiries not addressed by the repository, the first author can be contacted via email at luise.pruessner@psychologie.uni-heidelberg.de.
